# The Development of Micromachined Gyroscope Structure and Circuitry Technology

**DOI:** 10.3390/s140101394

**Published:** 2014-01-14

**Authors:** Dunzhu Xia, Cheng Yu, Lun Kong

**Affiliations:** Key Laboratory of Micro-Inertial Instrument and Advanced Navigation Technology, Ministry of Education, School of Instrument Science and Engineering, Southeast University, Nanjing 210096, China; E-Mails: 101010203@seu.edu.cn (C.Y.); lunkong2013@163.com (L.K.)

**Keywords:** micromachined/micro gyroscopes, gyroscope structure, gyroscope circuitry, Coriolis effect, Sagnac effect, gyroscope review

## Abstract

This review surveys micromachined gyroscope structure and circuitry technology. The principle of micromachined gyroscopes is first introduced. Then, different kinds of MEMS gyroscope structures, materials and fabrication technologies are illustrated. Micromachined gyroscopes are mainly categorized into micromachined vibrating gyroscopes (MVGs), piezoelectric vibrating gyroscopes (PVGs), surface acoustic wave (SAW) gyroscopes, bulk acoustic wave (BAW) gyroscopes, micromachined electrostatically suspended gyroscopes (MESGs), magnetically suspended gyroscopes (MSGs), micro fiber optic gyroscopes (MFOGs), micro fluid gyroscopes (MFGs), micro atom gyroscopes (MAGs), and special micromachined gyroscopes. Next, the control electronics of micromachined gyroscopes are analyzed. The control circuits are categorized into typical circuitry and special circuitry technologies. The typical circuitry technologies include typical analog circuitry and digital circuitry, while the special circuitry consists of sigma delta, mode matching, temperature/quadrature compensation and novel special technologies. Finally, the characteristics of various typical gyroscopes and their development tendency are discussed and investigated in detail.

## 1. Introduction

Micromachined gyroscopes are a kind of inertial sensors which are used to measure angular rate or attitude angle. Compared to traditional gyroscopes, micromachined gyroscopes have many advantages such as small size, light weight, low cost, high precision and easy integration *etc.* As a result, they are widely applied in many fields, including automotive applications for ride stabilization and rollover detection; some consumer electronic applications, such as video-camera stabilization, virtual reality, and inertial mice for computers; robotics applications; a wide range of military applications and so on [1]. Micromachined gyroscopes include the micromechanical and electronic parts which are achieved on either a single chip [2,3] or on two separate chips. The single chip integrated micromachined gyroscopes have the advantages of reducing the size and interface noise between the mechanical and electronic part. However, they need more advanced fabrication and package technology, and the cost is higher. Implementation on two separate chips has the advantages of lower cost, fabrication and package simplification, and eases optimization of the mechanical and electronic parts, respectively. However, this implementation is sensitive to outside interference, which decreases the gyroscope accuracy. The batch production with low cost and high precision is a target in the future.

The first micromachined gyroscope was described by the Draper Laboratory in 1988 [4], and then different kinds of micromachined gyroscopes emerged rapidly, such as MVGs, PVGs, SAW gyroscopes, BAW gyroscopes, MESGs, *etc.* [5]. Various principles, structures, and processes of micromachined gyroscopes are introduced in [6]. They can be fabricated in bulk micromachining, wafer bonding, surface micromachining, electroplating, Lithographie Galvanoformung Abformung (LIGA) and combined surface-bulk micromachining. Specially, the emergence of combined surface-bulk micromachining makes it easy to achieve single chip gyroscopes, in which the mechanical and electronic parts are integrated with high sensitivity and low noise in small size [7].

A micromachined gyroscope is usually a micro-resonator with two resonance modes, the primary mode and the secondary mode. The resonator can vibrate at its primary resonance mode with a constant frequency and amplitude by electrostatic, electromagnetic, piezoelectric or other force [8]. The angular rate or angle in the secondary mode direction can be detected because of the Coriolis force coupling between the two modes. So, electronics of the two modes is an important part as well. A perfect circuitry can make up for any fabrication imperfections and increase the robust immunity to the environment interference. Both PCB and ASIC technologies are used in MEMS gyroscope system. Compared to PCB implementation, ASIC implementation has the advantages of lower power, smaller volume, higher performance, and ease to mass production. As a result, ASIC implementation in micromachined gyroscope will be the main trend in the future.

Various micromachined gyroscopes had been reported. In 1998, a review of silicon micromachined accelerometers and gyroscopes was represented in [1]. Different types of micromachined gyroscopes were discussed including their design, operation and performance. However, interface electronics and packaging issues for micromachined gyroscopes were only briefly described. In [5], a detailed review of micromachined gyroscopes was reported in 2009. Different categories of gyroscopes were discussed and their key technologies were pointed out. However, the structures and circuits of micromachined gyroscopes have been changing rapidly in recent years. In this review, we will mainly focus on the recent micromachined gyroscopes, and circuitry technologies. Micromachined gyroscopes are categorized into MVGs, PVGs, SAW gyroscopes, BAW gyroscopes, MESGs, and MSGs. The control circuits of micromachined gyroscopes are categorized into typical circuitry and special circuitry. The typical circuitry technologies include the analog circuitry and digital circuitry, while the special circuitry technologies include the sigma delta, mode matching, temperature compensation and quadrature compensation and some other special circuitry technologies.

## 2. Micromachined Gyroscope Development

### 2.1. Micromachined Gyroscopes Principles

**Figure 1.**
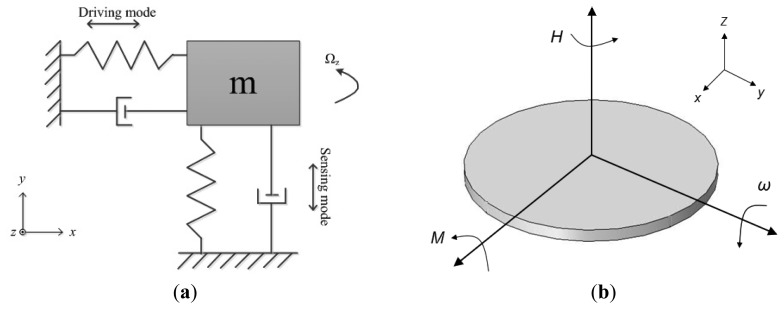
Principles of micromachined gyroscopes. (**a**) Coriolis effect. (**b**) Precession principle.

Micromachined gyroscopes are actually based on Coriolis effect or precession principle. Figure 1a shows the typical mechanics model of Coriolis effect gyroscopes. The proof mass **m** is supported by two springs and two dampers, equivalently [9]. Assume that the x*-*axis is the driving direction, y-axis is the sensing direction. When the proof mass works under simple harmonic vibration by applying an electrostatic, piezoelectric, electromagnetic or electrothermal force [10], the displacement along x-axis is

(1) $$x\left(t\right)=A_{x}\text{cos}(\omega_{x}t)$$

where *A_x_* is the amplitude, *ω_x_* is the driving angular frequency. When there is an angular rate Ω_z_ input rotation around the z-axis, this will cause Coriolis acceleration along y-axis:

(2) $$a_{y}=2\Omega_{\text{z}}\times \text{dx}/\text{dt}=-2\Omega_{\text{z}}A_{x}\omega_{x}\;\text{sin}\;(\omega_{x}t)$$

The proof mass will vibrate along y-axis because of the Coriolis force. The input angular rate Ω_z_ can be calculated by detecting the y-axis displacement. When the drive mode and sense mode are fully matched, *i.e.*, *ω_x_* = *ω_y_*, the responsive amplitude along y-axis achieves the maximum, while the bandwidth achieves the minimum one. In general, drive mode and sense mode should be matched for optimized sensitivity and bandwidth.

The conservation of angular momentum is shown in Figure 1b. The micromachined gyroscope based on precession principle usually has a rotor which is rotating around the spin axis (z-axis) at a constant speed to maintain an angular momentum **H**. When an angular rate orthogonal to the spin axis is applied, such as around y-axis, a precession moment **M** of the rotor is generated around x-axis by the equation:

(3) M=ω×H

This moment **M** causes the spin axis of the rotor to make a precession around the y*-*axis.

**Figure 2.**
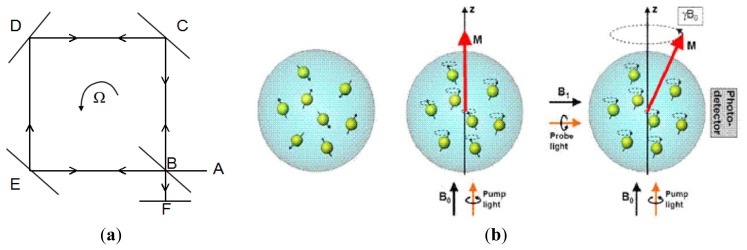
(**a**) Sagnac effect. (**b**) NMRG principle.

The micro optical gyroscopes are based on Sagnac effect. The basic principle of Sagnac's interferometer is given in Figure 2a. A light beam coming from source A is splitted by B into a beam in a clockwise (CW) direction BEDCB and another beam in a counterclockwise (CCW) direction BCDEB. The two beams are reunited at B and the interference fringes are observed in F. They will reach at F at the same time if the ring interferometer is static so the fringe shift is zero. When the ring interferometer with light source and fringe detector rotates with an angular Ω °/s, the fringe shift Δ*ϕ* is created in the interference pattern which is given by:

(4) $$\Delta \phi =\frac{8\pi S\Omega }{\lambda c}$$

where *S* is area enclosed by the light path, Ω is the rotation frequency, λ is the wavelength and *c* is the light velocity, respectively [11].

The principle of nuclear magnetic resonance gyroscopes (NMRGs) is to measure a corresponding shift in the Larmor precession frequency where the nuclear spins is applied in magnetic field. As shown in Figure 2b, when a static magnetic field B_0_ is applied, the magnetic moments will rotate about the direction of B_0_ at the Larmor precession frequency ω_L_:

(5) $$\omega_{L}=\gamma B_{0}$$

where γ is a constant of the gyroscope magnetic ratio depending on the type of material. When the NMRG is rotating about z-axis of the static field at an angular rate Ω, the measured Larmor frequency ω is shifted:

(6) $$\omega =\gamma B_{0}-\Omega$$

where γ and *B_0_* are known, the angular rate can be measured by monitoring the Larmor frequency [12].

### 2.2. MVGs

MVGs constitute one part of the fastest growing products in the micromachined gyroscope market. The application of these devices is rapidly expanding from automotive field to consumer electronics field and personal navigation systems. Small size, light weight, and low power consumption make MVGs ideal for use in handheld applications. MVGs have broken into the high precision market since their bias stability has reached 0.1°/h [13]. There are lots of categories about MVGs such as single mass, dual-mass and multi-mass gyroscopes, decoupled and coupled gyroscopes, single axis and multi-axis gyroscopes, angle (or type I) and angular rate (or type II) gyroscopes [14], electrostatic, piezoelectric, electromagnetic and electrothermal driving gyroscopes, dual-degree of freedom (DoF) and multi-DoF gyroscopes *etc.* It is difficult to get better performances with coupled MVGs since the drive oscillation and sense oscillation both act on a single mass, which makes mechanical coupling a serious problem [15]. In order to overcome the deficiencies, especially the mechanical coupling error, decoupled gyroscopes have become a main trend in recent years. Among MVGs, tuning fork gyroscopes (TFGs) and decoupled gyroscopes have attained very high precision and some of them can even reach tactical grade.

#### 2.2.1. TFGs

**Figure 3.**
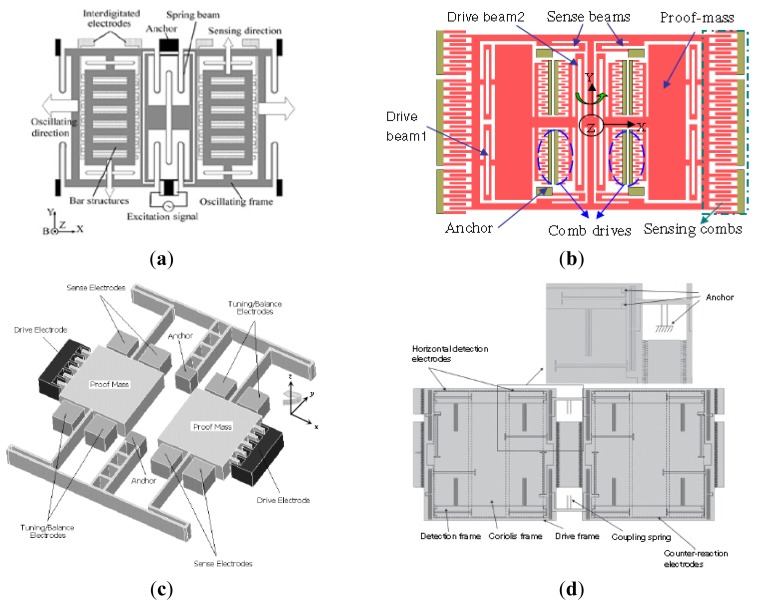

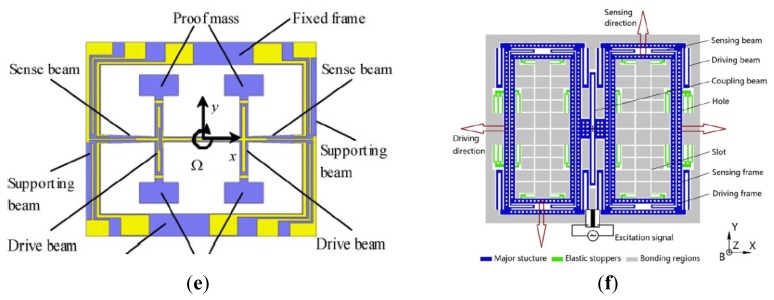
Various TFGs. (**a**) SIMIT. (**b**) PKU. (**c**) Georgia Tech. (**d**) CEA-LETI. (**e**) National University of Defense Technology (NUDT). (**f**) SIMIT.

A variety of MVGs have been developed over the past years, most of which belong to the tuning fork vibratory type employing differential excitation and detection mechanisms. The TFGs which adopt electrostatic driving and capacitive detection methods have relatively high precision. Any in-phase rotation, perpendicular to the drive mode, will then excite the out-of-plane rocking mode of the structure. Therefore, a large number of research institutions have been focusing on the TFGs. Various TFGs are shown in Figure 3.

A TFG with high Q-factor proposed by Shanghai Institute of Microsystem and Information Technology (SIMIT) and Shanghai Jiao Tong University (SJTU) which can work at atmospheric pressure is shown in Figure 3a [16,17]. It has two silicon oscillating frames, each of which is anchored on a glass substrate by four spring beams and they are connected to each other through a connection ring. The oscillating frames as well as the proof masses with bar structure can move above the glass substrate along the x or y direction. The bar structure electrodes and fixed comb finger electrodes on the glass substrate form the detection capacitors. The silicon surface is covered by an insulation layer, on which aluminum is deposited and patterned to form driving wires on the central spring beams. The experimental sensitivity is 6 mV/°/s and non-linearity is less than 0.5% for this gyroscope at atmospheric pressure was reported in 2005. Then, the TFG was improved by SIMIT in [18]. The resonant frequencies and the quality factors for drive and sense modes are 2.873 kHz and 2.989 kHz, 804 and 789 at atmospheric pressure, respectively. The nonlinearity of the gyroscope is 0.43%.

Figure 3b shows the lateral-axis TFG reported by Peking University (PKU) in 2009 [19]. The overall structure is symmetric with respect to both x*-*axis and y*-*axis. The drive combs and spring beams of the driving mode are located in the middle, surrounded by the comb fingers and spring beams of the sense mode. There are four folded sensing beams which support the entire movable parts and function as a torsional spring for the out-of-plane rotational motion. The two proof masses of the TFG are electrostatically actuated to vibrate oppositely along the x-axis. When a y-axis angular rate is applied to the gyroscope, Coriolis acceleration will be induced and the two proof masses will vibrate out of phase along the z-axis, which in turn will cause an out-of-plane rotational vibration of the moveable structure with respect to the y-axis. This out-of-plane vibration will be differentially picked up by two sets of vertical comb fingers. This z-sensing design has a relatively high Q-factor, so this gyroscope can work at atmospheric pressure. This TFG design also has a sensitivity of 2.9 mV/°/s in a full range of 800°/s with a nonlinearity of 0.9% and the noise floor of $0.035{}^{\circ}/\text{s}/\sqrt{\text{Hz}}$. This TFG design also has very low coupling. One year later, a modified decoupled comb capacitors for TFGs similar to Figure 3b was reported [20], resulting in a nonlinearity of 0.6% with full scale of 1000°/s and a bias stability of 0.05°/s (1σ) for 30 min.

The TFG reported by Georgia Tech has the highest precision among the TFGs. Sharma *et al.* presented a high-Q (Q_drive_ = 84,000 and Q_sense_ = 64,000) in-plane silicon-on-insulator (SOI) TFG in Figure 3c [21,22]. In this design, the proof masses are driven at resonance along the x-axis, and the Coriolis acceleration induced by rotation around the z-axis is sensed capacitively along the y-axis. The drive and sense resonant modes are balanced electrostatically within 0.07% of each other and the measured rate results show a sensitivity of 1.25 mV/°/s in a bandwidth of 12 Hz. In 2006, the mode-matched tuning fork gyroscope (M^2^-TFG) displayed an overall rate sensitivity of 24.2 mV/°/s. Allan Variance analysis of the mode-matched device demonstrates an angle random walk (ARW) of $0.045{}^{\circ}/\sqrt{\text{h}}$ and a measured bias stability of 0.96°/h [23,24]. Two years later, Georgia Tech reported a TFG with bias drift as low as 0.15°/h and ARW of $0.003{}^{\circ}/\sqrt{\text{h}}$– the lowest recorded for a silicon MEMS gyroscope at that time. The maximum scale factor of the gyroscope is 88 mV/°/s and the microsystem bandwidth could be configured between 1 to 10 Hz [13,25]. The improvement of the Georgia Tech TFG can be seen in Table 1.

Figure 3d shows a 3D capacitive TFG designed by CEA-LETI, France [26]. Different from the TFG in [19], where the out-of-plane motion is detected by vertical electrodes of different heights with the disadvantages of poor linearity and impossible to generate trimming [27], the 3D gyroscope utilizes the out-plane sensing with suspended horizontal electrodes. The mobile structure is made within a 30 μm thick Si top layer of a SOI substrate, while poly-Si deposited on top of a sacrificial PSG layer serves as suspended top electrodes and connection wires. The biggest innovation is that a new technological process is proposed to enable 3D sensing with suspended horizontal electrodes for out-of-plane detection. Compared with conventional horizontal electrode process, this technology maintains lower parasitic capacitance in gyroscopes. Also, this technology provides a reference platform to manufacture 3D gyroscopes.

Different from conventional detection scheme, National University of Defense Technology (NUDT) proposed shear stress detection scheme which can simplify the electrodes fabrication of the sensor for the structure miniaturizaion. Moreover, the sense electrodes are needless to be divided into two parts on each sidewall anymore. As shown in Figure 3e, in order to increase the sensitivity of the sensor, the sense beam is designed to be a symmetric tapered beam. The resonant frequency in drive mode is 14.993 kHz and the Q factor is about 7600 at atmosphere pressure. The experimentally obtained scale factor is 23.9 mV/(°/s), the nonlinearity is 1.1% with full scale of ±150°/s and the noise floor is $0.1{}^{\circ}/\text{s}/\sqrt{\text{Hz}}$ [28]. Tongji University (TJU) and SIMIT proposed a TFG designed for high-g shock environments in 2013. As shown schematically in Figure 3f, the TFG consists of two symmetrical frame structures which are connected by middle coupling beams. Each part of the major structure is composed of four driving beams, four sensing beams, one driving frame and one sensing frame. The driving beams connect the driving frame with the bonding regions which are anchored on silicon substrate. The sensing frame is located on the inner of the driving frame, connecting with the driving frame by the sensing beams. The gyroscope was fabricated on a 300 μm thickness silicon wafer through bulk silicon micromachining technology. The working frequencies of the gyroscope in the drive and sense modes are 10,240 and 11,160 Hz, respectively. Shock experiments show that the shock resistance of the gyroscope along x-axis is 15,000 g, y-axis is 14,000 g and z-axis is 11,000 g [29]. Table 2 summarizes the TFGs mentioned above.

#### 2.2.2. Decoupled MVGs

**Figure 4.**
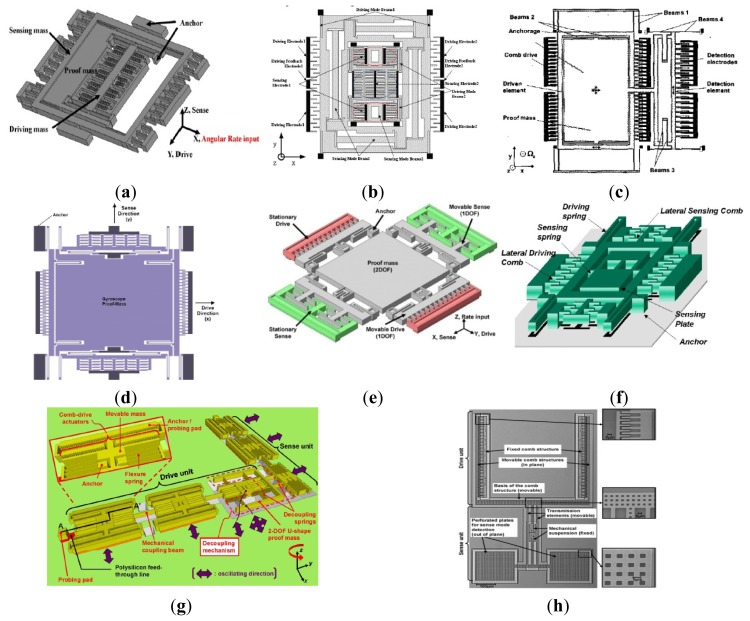
Various decoupled gyroscopes. (**a**) and (**b**) PKU. (**c**) HSG-IMIT. (**d**) UC Irvine. (**e**) Middle East Technical University. (**f**) Korean universities. (**g**) National Taiwan University. (**h**) Saarland University.

Quadrature signal has a major problem with regard to the gyroscope performance due to various cross-coupling mechanisms of the oscillation modes. Therefore, the quadrature signal is considered as the interfering signal (error signal) and unrelated to the external angular rate, which is not in phase to the effective signal from the sensing oscillation induced by the Coriolis forces. The quadrature error signal implicates a greater influence on temperature drift in gyroscopes which should be reduced by appropriate decoupling techniques. Another main reason for the need of eliminating the quadrature error signal is the improvement of the signal-to-noise ratio (SNR). Adopting decoupled mechanism, zero-rate-output (ZRO) and quadrature error are significantly reduced in the presence of structural imperfections. Moreover, the structure suppresses the effect of the parasitic force caused by fabrication imperfections and asymmetries. In order to improve the gyroscope performance and achieve tactical-grade application, various decoupled MVGs are developing recently, as seen in Figure 4.

A decoupled gyroscope of PKU is shown in Figure 4a. It has the structure of double decoupled lateral axis gyroscope [30]. The bar in the center is the driving mass, which is connected with the driving comb fingers. The large asymmetrical structure is the proof mass, while the outer frame is sensing mass, connected to sensing comb fingers. These three masses are jointed together by four groups of springs. Two groups of springs are torsional springs. Six anchors are arranged to fix the whole structure onto substrate, four of which are inside the asymmetrical proof mass while the other two are outside the frame. The sensitivity is 22 mV/°/s while the nonlinearity is 2.19% at atmospheric pressure. The noise floor is $0.02{}^{\circ}/\text{s}/\sqrt{\text{Hz}}$. Another novel lateral axis gyroscope with varying environmental parameters is shown in Figure 4b [31]. In this design, the vertical comb fingers are adopted to sense the out-of-plane motion. The outer frame and the inner frame with symmetrical comb fingers connect as the drive and sense element, respectively. Four folded cantilever beams and four vertical spring beams are employed to suppress the mechanical coupling between the two modes. The inner frame will make a vibration motion along z-direction, due to the Coriolis force, in case of the angular rate introduced around the y-axis. With the high quality factor and small coupling, the gyroscope can work well even at atmospheric pressure. The sensitivity and nonlinearity are 6.7 mV/°/s and 0.51% with full scale of 800°/s, respectively.

Figure 4c shows the doubly decoupled gyroscope of HSG-IMIT [32]. This gyroscope comprises four types of springs (beams 1 to beams 4) and three oscillators. Due to the arrangement of the springs, only the driven element and the proof mass are excited to a linear oscillation along the drive mode (x-axis) and the proof mass as well as the detection element (detection unit) execute the secondary oscillation (y-axis). In this way, the secondary oscillation does not influence the driving mechanism and parasitic effects of the comb drives and the primary oscillation are suppressed. The sensitivity and nonlinearity are 20 mV/°/s and 0.1% with scale factor of ±100°/s, respectively and the measured bias drift is ±0.5°/s. However, this double decoupled MVG does not represent a great improvement compared with the single decoupled gyroscope with the sensitivity of 10 mV/°/s, nonlinearity of 0.1% and bias drift of ±2.5°/s.

A novel MVG has been reported at UC Irvine that provides enhanced decoupling of the drive and sense modes, and increased actuation and detection capacitances beyond the fabrication process limitations in Figure 4d [33]. The decoupling mechanism aims to minimize the effects of fabrication imperfections and the resulting anisoelasticities, by utilizing independent folded flexures and constrained moving electrodes in the drive and sense modes. The gyroscope exhibits the sensitivity of 0.91 mV/°/s, excellent linearity, and a noise floor of $0.25{}^{\circ}/\text{s}/\sqrt{\text{Hz}}$ at 50 Hz bandwidth at atmospheric pressure. The structure of the decoupled gyroscope is symmetric and the parasitic force caused by the structural asymmetries is greatly suppressed.

Researchers from the Middle East Technical University in Turkey presented a symmetrical and decoupled micro gyroscope shown in Figure 4e. The fabricated gyroscope utilizes a standard three-layer polysilicon surface micromachining process (MUMPs) and nickel electroforming process in the early stage [34,35]. Afterwards, in 2005 they presented a single-crystal silicon symmetrical and decoupled (SYMDEC) gyroscope using a dissolved wafer [36], a high-performance SOI-MEMS gyroscope with decoupled oscillation modes was reported in 2006 and 2007 [37,38] and a 100-μm-thick single-crystal silicon MEMS gyroscope with an improved decoupling arrangement between the drive and sense modes in 2008 [39]. All the structures of the symmetrical and decoupled gyroscopes are similar to that in Figure 4e, with the differences of process technologies, materials, dimensions and packages. The scale factor is 22.2 mV/°/s, with a composite nonlinearity as small as ±0.6% within the ±50°/s measurement range. The zero-rate bias of the sensor is less than 0.1°/s after turn-on, while the bias stability is measured to be 14.3°/h. The rate equivalent white-noise density of the gyroscope is measured to be better than $6.9{}^{\circ}/\text{h}/\sqrt{\text{Hz}}$ in the recent report.

Researchers from Korean universities proposed another decoupled vertical MVG with an unbalanced inner torsion gimbal shown in Figure 4f [40,41]. The gyroscope has four driving springs supporting the whole mass, the driving comb electrodes, and the driving-sensing comb electrodes. Under the inner mass, there are the bottom electrodes that sense the tilting of the inner mass. The outer frame is connected to the substrate by four driving springs. The mass is divided into two parts, *i.e.*, the inner mass and the outer frame. The inner mass and the outer frame are connected with two torsional sensing springs. When the driving voltage is applied on the driving comb electrodes on the side of the outer frame, the mass oscillates along x-axis with the driving frequency. The gyroscope rotates around y-axis, which generates Coriolis force along z-axis. The generated Coriolis force makes the asymmetry inner mass tilt, which makes the capacitance between the inner mass and the bottom electrode change.

An interesting doubly decoupled gyroscope with a wide driving frequency range was reported by National Taiwan University (NTU) researchers [42]. As seen in Figure 4g, the novel feature of the gyroscope is the increased resonance bandwidths of both the drive and sense oscillators. This structure actually has several advantages. Firstly, the bandwidth enhancement ensures good frequency matching between the drive and sense oscillators despite any fabrication errors. Secondly, frequency tuning is not required. Thirdly, the gyroscope can be driven at any frequency within a ∼240 Hz bandwidth so that it easy to use. Finally, the doubly decoupled structure minimizes the coupling between the drive and sense modes. This gyroscope has a sensitivity of 4.28 mV/(rad/s).

Saarland University scientists proposed a decoupled surface micromachined gyroscope with a single-point mechanical suspension. As seen in Figure 4h, the gyroscope consists of seismic masses vibrating in anti-phase motion, and the spatial separation of the drive oscillator and the sense oscillator for decoupling between the drive mode and sense mode. The gyroscope has nice robustness to the fringe field effects, ambient pressure and temperature. The temperature coefficient of frequency is −45.3 ppm/K for the drive mode and −35.5 ppm/K for the sense mode at an ambient pressure of 3 mbar. The temperature coefficient of sensitivity is determined to be a good value of −858 ppm/K with constant drive amplitude. The noise equivalent resolution limit is 0.5°/s by using the existing non-optimized electronic unit and the sensitivity is 43.6 μV/°/s [43]. Table 3 lists a summary of TFGs mentioned above.

#### 2.2.3. Gimbal Gyroscopes

**Figure 5.**
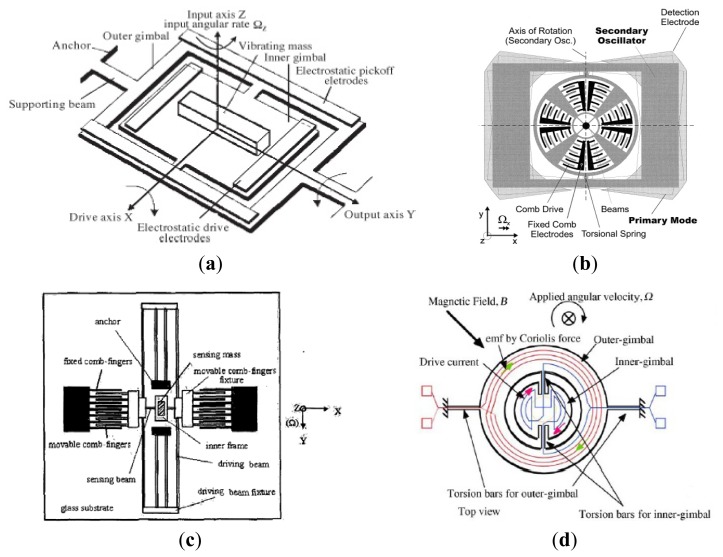
Various gimbal gyroscopes. (**a**) Draper Laboratory gimbal gyroscope. (**b**) HSG-IMIT MARS-RR. (**c**) SIMIT comb-gimbal gyroscope. (**d**) University of Hyogo gimbal gyroscope.

The gimbal micromachined gyroscopes that had been developed in inertial science was first reported by the Draper Laboratory with two gimbals [44], as shown in Figure 5a. The two-gimbals are supported by the torsional flexures. During the period of operation, the inner gimbal is driven at constant amplitude and frequency by the electrostatic drive electrodes. In presence of an angular rotational rate normal to the device plane, the Coriolis force will cause the outer gimbal to oscillate around its output axis with a frequency equal to the driving frequency and amplitude proportional to the inertial input rate. In 1997, a kind of two-gimbals micromachined gyroscope in China [45]. The device was fabricated using a quasi-LIGA process and consists of a vibrating mass, electrostatic drive electrodes, electrostatic pickoff electrodes, two anchors, supporting beams, an inner gimbal and an outer gimbal.

Considering the features of dual-gimbal gyroscope and comb gyroscope, HSG-IMIT described a new micromachined angular rate sensor with two rotary oscillation modes (MARS-RR) of small size, low cost, and high performance, as seen in Figure 5b [46]. The device configuration mainly consists of the comb drives, torsional springs, beams and an outer rectangular structure. The comb drive structure is electrostatically driven to a rotary oscillation around the z-axis. In presence of an angular along x-axis, the Coriolis force will cause the outer rectangular structure to a rotary oscillation because of the high stiffness of the inner wheel. Then, the oscillation around y-axis is capacitively detected by the sensing electrodes on the substrate. Researchers from Shanghai Institute of Microsystem and Information Technology also proposed a novel micromachined comb-gimbal gyroscope which was fabricated with silicon-glass wafer bonding and deep reactive ion etching (DRIE) technology [47], as shown in Figure 5c. The gyroscope is driven by electrostatic force along x-axis. In presence of an angular rate along z-axis, the sensing mass will vibrate by torsion along x-axis due to the Coriolis acceleration. The input angular rate is obtained from the measured change of a pair of differential capacitors between the inner frame electrode and electrode on the glass substrate. The MARS-RR and comb gimbal gyroscope has the advantage of improving resolution because drive mode and sense mode are decoupled well.

Figure 5d shows a MEMS gyroscope with double gimbal structure of University of Hyogo [48]. The gyroscope mainly consists of an inner gimbal with inner coils, outer gimbal with outer coils, torsion bars and permanent magnets. The inner gimbal is driven by a current in inner coils to oscillate at its own resonant frequency around the torsion bars, while the outer gimbal is steady because the vibration of the inner gimbal is parallel to the torsion bar for the outer gimbal. When an angular rate is applied perpendicularly to the plane, Coriolis force at the center mass makes the oscillation of the outer gimbal and the outer-coil provides an electromotive force for the voltage detection. The device can operate at atmospheric pressure because the device has no critical parts such as narrow gaps or comb drivers, and accurate alignment of the magnetic field. Table 4 shows the summary of decoupled gyroscopes mentioned above.

#### 2.2.4. Vibrating Ring Gyroscopes

**Figure 6.**
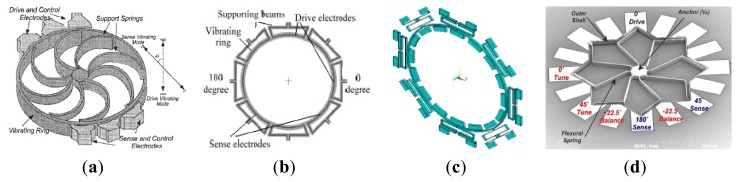
Various VRGs. (**a**) University of Michigan VRG. (**b**) and (**c**) Chinese Academy of Sciences VRG. (**d**) Georgia Tech RSG.

The vibrating ring gyroscope (VRG) provides a number of advantages, including excellent mode matching, high resolution, low ZRO, and long-term stability. Among the various silicon MVGs, researchers from University of Michigan first developed the vibrating ring gyroscope which is shown in Figure 6a [49–51]. This VRG consists of a ring, semicircular support springs, drive, sense and control electrodes. The ring is electrostatically vibrated into an elliptically-shaped primary flexural mode with the fixed amplitude. When the device is subjected to rotation, the Coriolis force causes energy to be transferred from the primary mode to the secondary flexural mode, which is located 45 degrees apart from the primary mode, making the amplitude build up proportionally in the latter mode. This build-up is capacitively monitored. The University of Michigan was the first institute using a single-wafer high aspect ratio p++/polysilicon trench-refill technology to design and fabricate VRG in 1998 [49]. In 2001, the vibrating ring gyroscope was fabricated through the high aspect-ratio combined poly and single-crystal silicon MEMS technology (HARPSS). An open-loop sensitivity of 200μV/°/s in a dynamic range of ±250°/s was measured under low vacuum conditions for a prototype device tested in hybrid format. The resolution for a prototype sensor with a quality factor of 1200 was measured to be less than 1°/s in 1 Hz bandwidth. Elimination of the parasitic capacitance and improvement in the quality factor of the ring structure will improve the resolution to $0.01{}^{\circ}/\text{s}/\sqrt{\text{Hz}}$ [50]. Afterwards, the performance is improved by being fabricated in oriented single-crystal silicon (SCS) with high Q (12,000), good nonlinearity (0.02%), large sensitivity (132 mV/°/s), low output noise ( $10.4{}^{\circ}/\text{h}/\sqrt{\text{Hz}}$) and high resolution (7.2°/h) in 2002 [51].

In recent years, researchers in Chinese Academy of Sciences (CAS) have been researching the VRGs. In 2010, a micromachined VRG with highly symmetric structure shown in Figure 6b was proposed to suit the harsh environments such as accelerations, ambient temperatures and so on [52] and then the Q-factor of the gyroscope in the air was improved in 2012 using feedback control [53]. The micromachined VRG consists of a ring with radius of 4 mm and eight “M” type beams to support the ring. There are four drive electrodes (at 0, 90, 180, 270 degrees) and four sense electrodes (at 45, 135, 225 and 315 degrees). The ring of the VRG has two elliptical-shaped identical flexural modes (drive mode and sense mode) with the same resonant frequency in different directions (45 degrees apart from each other mode). Because of the residual stress during fabrication, environmental interferences or other factors, there always exists a frequency split between these two modes in reality. The less the frequency split, the higher the sensitivity. Electromagnetic driving and inductive sensing were adopted to make the closed loop control easier. The ring of the micromachined VRG is vibrated with fixed amplitude at drive mode frequency applying the alternative current on the drive electrodes with the help of the magnetic field. When subjected to rotation around its normal axis, the vibrating mode of the ring will be transferred from the drive mode into the sense mode because of the Coriolis force. The oscillation amplitude of the sense mode, which is proportional to the rotation rate, will generate the induced voltage on the sense electrodes. The gyroscope has a sensitivity of about 8.9 mV/°/s. The measured nonlinearity is about 0.23% over the range of ±200°/s. The resolution of this gyroscope is about 0.05°/s.

The same year, an electrostatically actuated micromachined VRG with highly symmetric support beams as seen in Figure 6c was presented by researchers from the Chinese Academy of Sciences [54]. This gyroscope consists of a circular ring, 16 folded support springs outside the ring and 16 uniformly distributed electrode anchors inside the ring for drive, sense and control of vibration of the ring. Through the drive electrodes, the ring is electrostatically excited into an elliptically-shaped primary flexural mode with a fixed amplitude. When the gyroscope is subjected to rotation around its normal axis, the Coriolis force causes energy to be transferred from the primary drive mode to the secondary flexural sense mode, which is located 45 degrees apart from the primary mode, causing a build-up of oscillation amplitude proportional to the rotation rate in the latter mode. This build-up is capacitively monitored by a series of electrodes around the ring, and then the angular rate is obtained. The frequency split of the gyroscope can be adjusted from 160 Hz before balancing to less than 0.1 Hz after balancing, the Q-factor could achieve 22,000 in vacuum, the resolution of the gyroscope is 0.05°/s, and the measured non-linearity is 0.06% in the ±50°/s range. The micromachined VRGs designed by Chinese Academy of Sciences have several advantages: (1) the highly inherent symmetry of the structure makes them less sensitive to spurious vibrations; (2) Since two identical flexural modes of the structure with nominally equal resonant frequencies are used to sense rotation, the sensitivity of the sensor is amplified by the quality factor of the structure, resulting in higher sensitivity; (3) The VRGs are less temperature sensitive since the vibration modes are affected equally by temperature.

Zaman *et al.* from Georgia Tech reported a novel multiple-shell silicon resonating star gyroscope (RSG) which is formed as a merged superposition of two square shells, yielding in-plane flexural modes, as seen in Figure 6d [55]. The RSG consists of an eight-folded outer shell, which is anchored to a central post by means of eight flexural springs. These springs are designed to make a balanced device with two identical modes that have equal natural frequencies and are 45 degrees apart from each other. In order to alleviate the low Q operating mode with 65 μm thick trench-refilled polysilicon structural material using the HARPSS process, the RSG is fabricated in 40 μm thick SOI device layer using a simple two-mask process. The Allan deviation bias drift is 3.5°/h and the sensitivity is 16.7 mV/°/s. Compared with other VRGs, the RSG not only incorporates all the necessary advantages of the VRG, but also offers a 40% increase in the electrode area and overall resonant mass for a given radial geometry, enabling a better overall noise resolution and sensitivity of the system and making the RSG a better alternative for area constraints.

**Figure 7.**
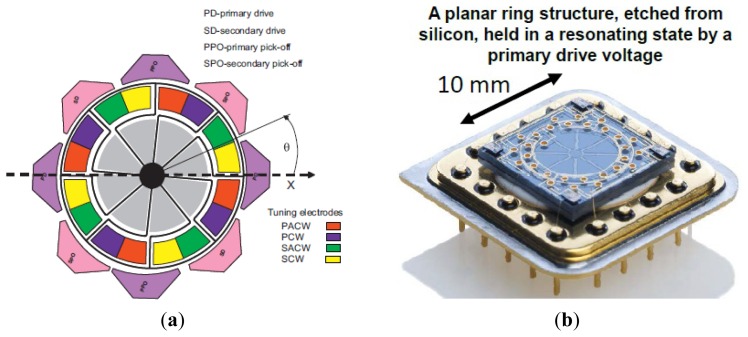
SiREUS.

SiREUS is the 3-axis MEMS Rate Sensor (MRS) developed by a UK consortium of SELEX Galileo (Edinburgh), SEA (Bristol) and AIS (Plymouth). SiREUS is the smaller, lighter and less power consuming space rad-hard gyro in the world until 2012. There is long history of SiREUS research from 1999 to 2013. SiREUS is actually an integrated ring gyroscope. Figure 7a illustrates the MEMS ring gyroscope used in the investigation proposed by Newcastle University. The vibrating gyroscope structure is a suspended ring with a radius of 8 mm, width of 203 μm and thickness of 155 μm. There are sixteen electrodes which provide electrostatic actuation, sensing and tuning of the modes. There is a 10 μm gap between the ring and the electrodes, which forms a nominal capacitance of 0.96 pF. Figure 7b shows the packaged gyroscope with metal that is used to provide the vacuum seal. The natural frequencies of the primary and secondary modes are 14.2545 kHz and 14.2563 kHz, respectively, with the Q-factors of both modes of the value 28,400. The reported test angular random walk is $0.009{}^{\circ}/\sqrt{\text{h}}$, bias over temperature is 10°/h, max constant angular rate bias drift is 0.1°/h/day and scale factor linearity <1,000 ppm [56,57]. Table 5 describes the summary of VRGs mentioned above.

#### 2.2.5. Multi-DoF(Degree of Freedom) MVGs

**Figure 8.**
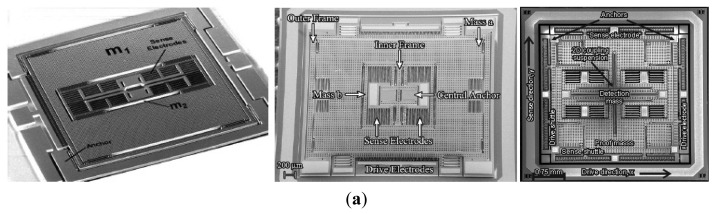

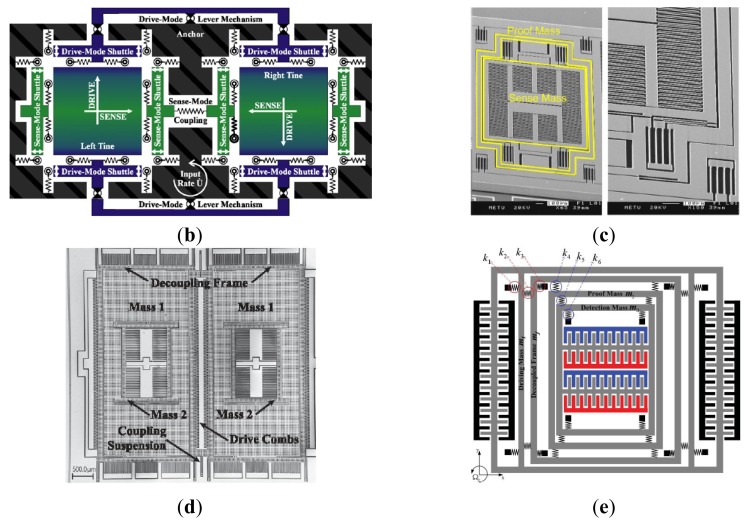
Various multi-DoF gyroscopes. (**a**) 2-DoF sense and 1-DoF drive gyroscope of University of California. (**b**) 1-DoF sense and 2-DoF drive gyroscope of University of California. (**c**) The 3-DoF gyroscope of Carnegie Mellon University. (**d**) The 6-DoF gyroscope of University of California. (**e**) The 4-DoF gyroscope of Harbin Engineering University.

In order to get the maximum possible gain, gyroscopes are designed to work at the peak of the resonant frequencies and the maximum possible gain can be achieved by matching the drive mode and sense mode. However, the system is very sensitive to the variations in system parameters when in the resonant frequencies. As we know, the gain is high after mode-matching, while the bandwidth is narrow under high quality factor conditions. To improve the robustness of the MVG, Shkel *et al.* presented a novel approach that aims to expand in expanding the design space of the device by increasing the DoFs of the system [58]. Afterwards, the various multi-DoF gyroscopes which can be seen in Figure 8 have developed rapidly.

Figure 8a shows three kinds of 3-DoF gyroscopes of University of California, Irvine with 1-DoF drive mode oscillator and 2-DoF sense mode oscillator that provides inherent robustness against structural parameter variations [59–62]. The 2-DoF sense mode oscillator provides a frequency response with two resonant peaks and a flat region between the peaks, instead of a single resonance peak as in conventional gyroscopes. These gyroscopes are nominally operated in the flat region of the sense mode response curve, where the amplitude and phase of the response are insensitive to parameter fluctuations. Furthermore, the sensitivity is improved by utilizing dynamical amplification of oscillations in the 2-DoF sense mode oscillator. Thus, the improved robustness to variations in temperature, damping, and structural parameters is achieved, solely by the mechanical system design. The sense mode response in the flat operating region is also inherently insensitive to the pressure, temperature, and dc bias variations *etc.* The rate sensitivity is 28 μV/°/s, ARW is $0.09{}^{\circ}/\text{s}/\sqrt{\text{Hz}}$ and bias instability is 0.08°/s in the detailed report. Another 3-DoF gyroscope with 2-DoF drive mode and 1-DoF sense mode is shown in Figure 8b. It is a dual mass vibratory MEMS z-axis rate gyroscope with a very high sense mode Q-factor of 125,000. The drive mode of the gyroscope is formed by the two tines forced into anti-phase motion. In order to avoid the dissipation of energy through the substrate due to linear momentum and torque imbalances, the new architecture prioritizes the quality factor of the sense mode by mechanical design, where the linearly coupled anti-phase sense mode is balanced in both the linear momentum as well as torque [63].

Researchers from Carnegie Mellon University reported a micromachined gyroscope design concept with the help of a 2-DoF sense mode to achieve a wide bandwidth without sacrificing the mechanical and electronic sensitivity and to obtain robust operation against variations under ambient conditions in Figure 8c [64]. The proposed design has a bandwidth and robustness comparable to a dynamic vibration absorber (DVA) design, with an advantage of higher electronic sensitivity. Moreover, it provides a bandwidth and sensitivity comparable to a 1-DoF gyroscope with the near-matched mode operation, thus adding the advantage of higher robustness. Finally, it eliminates the need for complex feedback electronics implemented in closed-loop sensing by employing an inherently robust mechanical structure. The sensitivity is 131 μV/°/s in full scale of ±100°/s and the bias stability and ARW of the gyroscope are 131°/h and $1.15{}^{\circ}/\sqrt{\text{h}}$, respectively.

Figure 8d shows a novel design concept that combines the robustness of multi-DoF sensing with the common mode rejection of tuning fork devices in the anti-phase driven 6-DoF gyroscope [65]. The proposed 6-DoF robust tuning fork is designed to be fabricated by using an in-house, wafer-scale SOI process, with two 3-DoF devices coupled in the drive mode. The prototypes of the design are characterized for both rotational and acceleration inputs. For acceleration loads, the device responds in a common mode resulting in a 75% reduction in amplitude for a differential signal, while for rotations, it responded in anti-phase mode with sensitivities of 1.687 μV/°/s and −1.887 μV/°/s. This 6-DoF gyroscope has the advantage of anti- acceleration inputs compared with the 3-DoF gyroscope above.

Harbin Engineering University (HEU) researchers proposed a MVG with 2-DoF drive mode and sense mode in 2012. As seen the schematic in Figure 8e, the gyroscope comprises driving mass, decoupled frame, proof mass and detection mass. The driving mass and the decoupled frame are suspended by flexural beams, which can only oscillate in the drive direction. The proof mass and the detection mass are suspended relative to the decoupled frame by flexural beams. The proof mass can oscillate together with the decoupled frame both in the drive direction and the sense direction. The gyroscope is doubly decoupled through the single-degree flexural springs to restrain the masses in a direction and the decoupled frame to isolate the vibration between drive mode and sense mode. The biggest innovation is that the gyroscope not only utilizes a fully coupled 2-DoF sense mode, but also a fully coupled 2-DoF drive mode. The gyroscope demonstrates a bandwidth of 190 Hz in the drive mode and a bandwidth of 300 Hz in the sense mode at the operational frequency 5.0 kHz [66]. Similarly, Riaz *et al.* used Metal-Multi User MEMS Processes (MetalMUMPs) to fabricate the 2-DoF drive mode gyroscope to improve the robustness with a larger bandwidth of 754 Hz [67]. Table 6 shows the summary of multi-DoF gyroscopes mentioned above.

#### 2.2.6. Multi-Axis Gyroscopes

**Figure 9.**
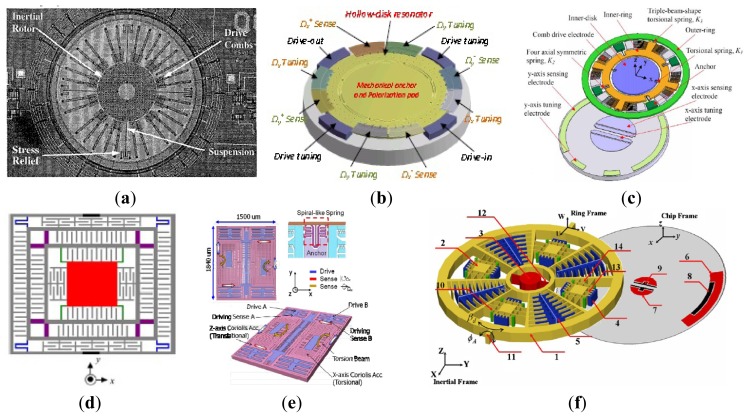
Various multi-axis gyroscopes. (**a**) UC Berkeley dual-axis gyroscope. (**b**) Georgia Tech dual-axis gyroscope. (**c**) National Tsing Hua University (NTHU) dual-axis gyroscope. (**d**) National Taiwan University (NTU) dual-axis gyroscope. (**e**) National Cheng Kung University (NCKU) dual-axis TFG. (**f**) NCKU tri-axis gyroscope.

In order to lower cost and increase efficiency in IMU, the dual-axis and multi-axis micro-gyroscopes are effective. Researchers of UC Berkeley presented a dual input axis vibrating wheel gyroscope which was fabricated by surface micromachining [68]. A 2 μm thick polysilicon disk with a 150 μm radius serves as an inertial rotor. As seen in Figure 9a, this inertial rotor is suspended 1.6 μm above the substrate by four symmetrically placed beams anchored to the substrate. The rotor is driven into angular resonance around the z-axis. When there is any angular rate of the substrate around the x/y-axis, a Coriolis angular acceleration about the y/x-axis will be induced. Then, a tilting oscillation of the rotor about the y/x-axis will occur. This device yields a random walk as low as $10{}^{\circ}/\sqrt{\text{h}}$ with cross-axis sensitivity ranging from 3% to 16% during open-loop operation. The random walk can be further improved to $2{}^{\circ}/\sqrt{\text{h}}$ by frequency matching at the cost of excessive cross-axis sensitivity.

Georgia Tech scientists reported a high-frequency single proof-mass dual-axis gyroscope which has been implemented using a revised version of the HARPSS process in Figure 9b [69]. This hollow-disk pitch-and-roll resonant single-proof-mass gyroscope has electrostatically tunable in-plane and out-of-plane resonance modes to enable mode matched operation at 0.9 MHz. To realize dual-axis (x-axis and y-axis) rate sensitivity, the device is designed to utilize an in-plane elliptical drive mode and two orthogonal out-of-plane sense modes. The scale factors for x and y-axis rotation rate are 127.4 μV/°/s/electrode and 213.8 μV/°/s/electrode with cross-axis sensitivity of 25.2% and 20.1%, respectively. The bias drift by Allan variance is 0.18°/s and 0.30°/s for x and y-axis mode, respectively.

Researchers from National Tsing Hua University, National Taiwan University and National Cheng Kung University have a series of investigations on multi-axis gyroscope. A dual-axis sensing decoupled gyroscope of National Tsing Hua University is shown in Figure 9c [70]. The main structure, which consists of three proof masses, can measure the angular rate of two different axes independently. A triple-beam-shape torsional spring is used to suppress the undesired in-plane linear motion of the proof mass. The capacity is used to measure the dual-axis angular rates. With the dc tuning voltages of 42 V and 54 V, respectively, the frequencies of the dual-axis sense modes are identical with the driving one. With the driving voltage of 20 V and the quality factor of 2000, the sensitivities of the dual-axis sense modes can reach 7.4 fF/°/s and 19.4 fF/°/s, respectively, and the nonlinearity of the dual-axis sense modes are only 0.04% and 0.29% with full scale of ±150°/s.

Figure 9d shows a CMOS-MEMS single-chip dual-axis gyroscope [3]. This gyroscope has integrated electrical and mechanical components to perform functions of sense, drive, and control on a single chip using TSMC 0.18 μm 1P6M process. Thus, the output of MEMS devices can be processed by CMOS circuits to reduce parasitic capacitance and noise. The comb fingers are divided into three groups, *i.e.*, top, middle, and bottom group. The middle comb fingers are responsible for driving the proof mass in the z-direction. If an angular velocity about the x-axis is applied, the proof mass will experience a Coriolis force in the y direction to be sensed by the bottom comb fingers. Similarly, when an angular velocity about the y-axis is applied, the proof mass will experience a Coriolis force in the x direction to be sensed by the top comb fingers electrodes. The sensitivity of angular velocity in the x and y directions are 0.087 mV/°/s and 0.017 mV/°/s, respectively.

Figure 9e shows an integrated dual-axis TFG (DTFG) designed by one research group of National Cheng Kung University [71]. The DTFG is fabricated by high-aspect-ratio silicon-on-glass (SOG) process and vacuum packaged by glass frit bonding. Furthermore, a CMOS drive/readout ASIC chip, which is fabricated by a 0.25 μm 1P5M standard CMOS process, is integrated with the fabricated DTFG by directly wire-bonding. The mechanical element of dual-TFG consists of two symmetric vibrating frames, which are driven to oscillate along the positive and negative y-axis reciprocally by the resonator drive electrodes, Drive A and Drive B. For drive mode operation, two symmetric vibrating frames are excited to oscillate about y-axis in the opposite direction. The corresponding motion of the two symmetric frames is detected by the sense electrodes, A and B in drive mode. In fact, the resonator drive/sense electrodes are the variable-area comb fingers. Therefore, the drive force and sense current are linear independent of the displacement of the frames. Besides, each vibrating frame comprises two seismic proof masses which are used to detect the z-axis and x-axis angular rates respectively. The rate sensitivities of z-axis and x-axis sense modes are 1.47 mV/°/s and 0.18 mV/°/s, respectively. The associated linearities are 0.9995% and 0.9996%. The noise-floors are $0.030{}^{\circ}/\text{s}/\sqrt{\text{Hz}}$ and $0.247{}^{\circ}/\text{s}/\sqrt{\text{Hz}}$ for z-axis and x-axis sense modes respectively.

Another research group at National Cheng Kung University first reported a wheel-like micromachined tri-axis gyroscope in Figure 9f [72–74]. The presented micro-gyroscope is mainly fabricated by the SOI technique operates exactly and the three-axis angular rates are capable of being detected. The outer-ring is driven by the rotational comb electrodes to rotate, within a limited interval, counterclockwise and clockwise alternatively around the z-axis. Once the micro-gyroscope is perturbed by Coriolis acceleration resulting from external rotation excitation around the y-axis, the outer ring responds to tilt in the direction of the x-axis. On the other hand, the inner-disc is forced to oscillate about the y-axis if the external rotation excitation is about the x-axis. All the tilts along x-axis or y-axis will result in the change of voltage output across the corresponding capacitors. Similarly, if the external angular excitation is about the z-axis, then the distributed translational proof mass will move in the radial direction and be detected by the comb electrodes. According to the report in 2010 [74], the scale factors of the tri-axis micro-gyroscope are 50.4 μV/°/s, 60.3 μV/°/s, and 71.2 μV/°/s for x, y and z-axis, respectively. The resolutions are about $0.72{}^{\circ}/\text{s}/\sqrt{\text{Hz}}$, $143{}^{\circ}/\text{s}/\sqrt{\text{Hz}}$ and $0.42{}^{\circ}/\text{s}/\sqrt{\text{Hz}}$ for x, y and z-axis, respectively. The cross-axis sensitivities are 22%, 9% and 1.84% for x, y and z-axis, respectively. S/N ratios reach 59.3, 13.8, and 140.1 for x, y and z-axis, respectively. Table 7 summarizes the multi-axis MVGs mentioned above.

#### 2.2.7. Vibratory Angle Gyroscopes

**Figure 10.**
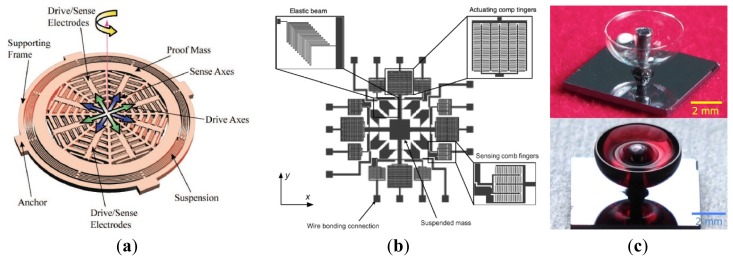
Various angle gyroscopes. (**a**) UC Irvine. (**b**) University of Minnesota. (**c**) University of Michigan.

MVGs can be classified into two basic types according to the measured physical quantity: angle gyroscopes and rate gyroscopes. Most of the reported gyroscopes are rate gyroscopes while angle gyroscopes are seldom reported. As seen in Figure 10a, an angle gyroscope proposed by Shkel consists of a mass connected to a suspension system, which is then in turn rigidly connected to a substrate below via anchors. The suspension has isotropic stiffness with identical principal axes of elasticity that it has the same stiffness in all directions. The device is actuated electrostatically using fixed parallel plate electrodes anchored to the substrate below and interwoven throughout the mass. The gyroscope uses transimpedance configuration for velocity detection and an additional cascaded integrator for position detection [13,75]. To realize this angle gyroscope, Park from Sejong University presented an adaptive control algorithm which uses a trajectory following approach and the reference trajectory so that rotation angle can be directly measured without integration of angular rate, thus eliminating the accumulation of numerical integration errors [76]. Researchers from National Chiao Tung University proposed another method, which does not need to measure both the positions and velocities, to directly measure the rotation angle. The proposed method is based on state estimation techniques. The system parameter estimation is skillfully arranged so that it can be done using various existing state observer algorithms. The algorithm compensates different types of imperfection even when the proof mass of a gyroscope is unknown [77].

University of Minnesota researchers presented an innovative design for a vibrating gyroscope that can directly measure both angle and angular rate [78]. As seen in Figure 10b, the gyroscope is based on DRIE patterning on silicon on insulator (SOI) technology. The design is based on the principle of measuring the angle of free vibration of a suspended mass with respect to the casing of the gyroscope. Two transverse comb actuators are used to provide forces in x and y direction. The gyroscope has a central vibrating mass which is connected to the actuation and sensing comb structures through eight springs. The drive and sense comb drives are rigidly connected to the vibrating mass. Shao *et al.* from Georgia Tech proposed a fabricated silicon dioxide μHSR coated with 30 nm of ALD TiN in 2012. The μHSR is electrically excited by one of surrounding electrode and the resonance is picked off from shell through the substrate [79]. University of Michigan scientists proposed a rate-integrating gyroscope using high-Q materials based on 3-D fabrication process. As seen in Figure 10c, the gyroscope has high aspect ratio (height/radius > 1) 3-D hollow structures (thickness < 200 μm) with ultra-smooth surface roughness. The structural asymmetry and small damping of the fabricated birdbath resonator make it promising for micro rate-integrating sensor [80].

### 2.3. PVGs

**Figure 11.**
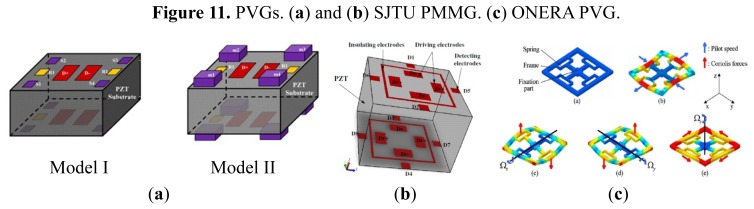
PVGs. (**a**) and (**b**) SJTU PMMG. (**c**) ONERA PVG.

Different from the conventional micromachined vibratory gyroscope, which has the structure of suspending springs and proof masses, the piezoelectric vibrating gyroscopes have no such moving part as a whole. Therefore, the piezoelectric vibrating gyroscopes have prominent robustness, wide measuring range and higher resistance to outer shock and shake. They can work in atmospheric environment and have no special requirement of vacuum packaging. Among the piezoelectric vibrating gyroscopes, they can be simply classified into two categories according to their shape: the solid-state gyroscopes and the plane gyroscope, as seen in Figure 11a,b respectively.

In recent years, solid vibratory gyroscopes with high resistance to shock and shaking, and with wide measuring ranges have received more attention. In 2006, a simple solid vibratory micro-gyroscope using the 29th resonance mode as the reference vibration [81] was proposed by Maenaka *et al.* of University of Hyogo, Japan. Then in 2009, Wu *et al.* of SJTU improved the device on lumped mass and dual-axis detection which is named the piezoelectric micromachined modal gyroscope (PMMG) in [82], as seen in Figure 11a, including two models. Model I uses the 7th resonance mode as the working resonance mode while Model II uses the 6th resonance mode. Although the higher order resonance mode movements of the PZT prism mass elements are nearly in one direction, there are undesired movements in other directions which would result in coupling effects and errors even when the Poisson effect is considered. To further improve the sensitivity of the gyroscope, the effect of driving method on reference vibration is taken into account. Researchers have invented several novel structures of single axis and double axis gyroscopes reported in detail in [83]. The simulation results of the measured angular velocity for single axis and double-axis gyroscopes are 4.530875 mV/°/s and 0.927817 mV/°/s, respectively. The experimental results need more challenges to detect such small variable values in the PMMG region in the future. The university also proposed a dual-axis PVG with a very simple structure, high resistance to heavy acceleration or shock and a low cost, as shown in Figure 11b. The complicated movement of the PZT prism, which could not be equivalent to the mass–spring system, is analyzed by the FEM. The optimal structure size is 4 × 4 × 3 mm^3^ [84].

Roland *et al.* presented a piezoelectric MEMS Coriolis Vibrating Gyroscope based on a single GaAs vibrating structure allowing the measurement of rotation rate along 3 orthogonal sensitive axes, as seen in Figure 11c [85,86]. The gyroscope is a deformable square frame which is connected by symmetric crossing springs to an anchor located at its center. The drive mode consists of an in-plane vibration of the frame, *i.e.*, two opposite sides of the frame bend inwards when the other two bend outwards. The x-axis sense mode is an out-of-plane vibration of two opposite sides of the frame: one bends upwards while the other bends downwards; the y-axis sense mode is a similar vibration using the two other sides of the frame. The z-axis sense mode is an in-plane bending of the fixed beams which strain the square frame into a rhombus. The sensitivities for each of its sensitive axis are 1.6 × 10^−16^ C/°/s.

### 2.4. SAW Gyroscopes

**Figure 12.**
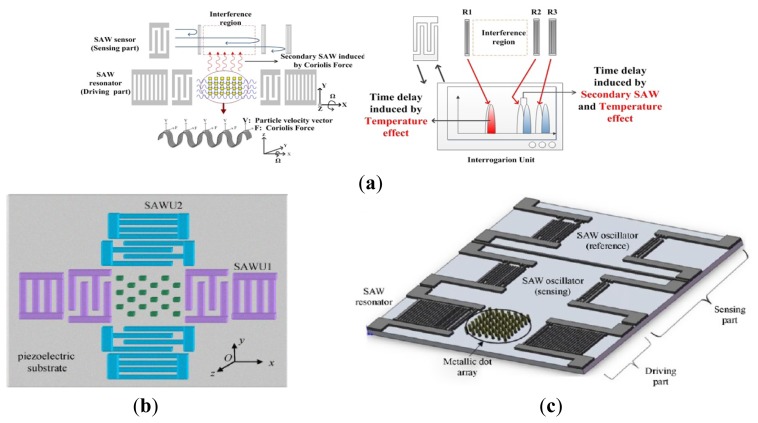
SAW gyroscopes. (**a**) A new kind of single axis SAW gyroscope. (**b**) A novel MEMS IDT dual-axes SAW gyroscope.

Like piezoelectric vibrating gyroscopes, surface acoustic wave (SAW) gyroscopes do not have any suspended structures so that they have been greatly focused on owing to their superior inherent shock robustness, wide dynamic range, and low power consumption. Many groups have reported SAW-based gyroscopes using different designs and operating principles. In recent years, researchers from Ajou University and Chinese Academy of Sciences represented a new kind of SAW gyroscope with an advantage of no battery requirement to operate sensing systems [87–90]. The schematic diagram and working principle of the SAW gyroscope are shown in Figure 12a. It consists of a two-port SAW resonator (driving part) with a metallic dot array, and a SAW sensor (sensing part) structured by a reflective delay line with three reflectors. The SAW resonator forms a standing wave pattern between the two interdigital transducers (IDTs), where the metallic dots of mass are aligned onto the anti-nodes of the standing wave. The metallic dots at such position experience large vibration in the z-axis. When there is an input angular rate in the x-axis, a Coriolis force will occur in the y-axis. The vibration induced by this Coriolis force serves as the driving vibration motion for this gyroscope. The longitudinal SAW generated by this Coriolis force is propagated to the interference region. This secondary SAW interferes with the Rayleigh SAW excited by the sensor IDT and propagated along the x-axis. The interference changes the acoustic velocity of the Rayleigh SAW and induces a time delay as seen (the right one). By measuring the time delay of the reflected SAW signal, the input rotation can be evaluated. The obtained sensitivity is approximately 1.23°/(°/s) in an angular rate range of 0–2,000°/s according to the latest report. In 2012, two single axis SAW gyroscopes on silicon substrates were used to measure the angular velocities of dual-axis [91]. This dual-axis sensor uses the progressive wave instead of the standing wave so that the external circuit configuration is simple and additionally it could be easily implemented with low cost. The sensitivity and linearity of the SAW gyroscopes for y-axis are 45.32 and 0.907 Hz/°/s, respectively. The sensitivity and linearity for the *x*-axis are 27.34 and 0.837 Hz/°/s, respectively.

Using two single axis SAW gyroscopes to measure the angular velocities of dual-axis, will enlarge the size and increase the cost. To solve these problems, Liu *et al.* proposed a novel MEMS IDT dual-axis SAW gyroscope to detect two orthogonal angular velocities with the advantages of small-size, low-cost, rugged to shock and ease to be fabricated [92], as shown in Figure 12b. Different from the single axis gyroscope in the configuration, the dual-axis SAW gyroscope replaces the SAW sensor in the single axis one with another SAW resonator. The two resonators in the dual-axis gyroscope are the surface acoustic wave unit 1 (SAWUl) and unit 2 (SAWU2), respectively. If the angular velocity along the x-direction is to be sensed, the SAWUl as a resonator will generate the primary standing SAWs along the x-direction, while the SAWU2 as a sensor will measure the secondary SAW along the y-direction and outputs a voltage, according to which the x-direction angular velocity can be gained. On the contrary, SAWU2 works as a resonator while SAWUl works as a sensor when sensing the angular velocity along the y-direction.

Ajou University researchers presented a novel SAW-based gyroscope with an 80 MHz central frequency which was developed on a 128° YX LiNbO_3_ piezoelectric substrate. As shown in Figure 12c, the sensor is composed of a SAW resonator, metallic dots, and two SAW delay lines. The SAW resonator is used to generate a stable standing wave with a large amplitude, the metallic dots are used to induce a Coriolis force and to form a secondary SAW, and two delay lines are formed to extract the Coriolis effect by comparing the resonant frequencies between these two delay lines. The sensitivity is approximately 172 Hz/(°/s) at an angular rate range of 0–500°/s with good thermal and shock stabilities [93]. Table 8 shows the summary of SAW gyroscopes mentioned above.

### 2.5. BAW Gyroscopes

**Figure 13.**
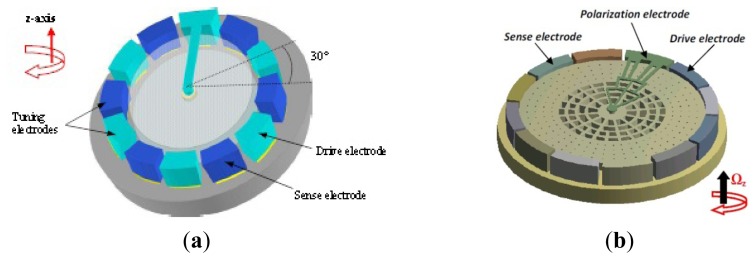

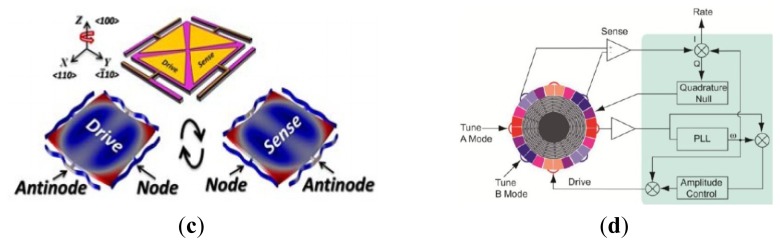
BAW gyroscopes. (**a**), (**b**) and (**c**) Various BAW gyroscopes of Georgia Tech. (**d**) UC Davis.

Normally, MVGs work at low frequencies (3–30 kHz) and rely on the increase in the mass and excitation of the driving amplitude to reduce the noise floor and improve the bias stability. However, increasing the mass and driving amplitude are difficult to achieve relatively low power and small size. Thus, increasing the resonant frequency and Q are significant by utilizing bulk acoustic modes that are of less thermoelastic damping (TED) compared with the flexural modes. In order to decrease the noise floor, researchers from Georgia Tech presented 800 μm diameter center-supported single crystal silicon (SCS) bulk acoustic wave (BAW) gyroscopes operating in high order elliptical modes at 5.9 MHz [94], as seen in Figure 13a. The BAW gyroscope is fabricated on 50 μm thick SOI using the high aspect ratio combined polysilicon with single crystal silicon (HARPSS) process to obtain 250 nm capacitive gaps and exhibited ultra high Q in excess of 200,000. There are several modes which are used as the driving and sense mode for the choice of the degenerated mode frequencies. Only the higher order elliptical modes of 800 μm diameter SCS BAW gyroscope that are spatially 30 degree apart have identical frequencies. The primary elliptical modes are different. Increasing the BAW gyroscope diameter to 1,200 μm, the primary elliptical modes are identical to suit device operation in [95]. The gyroscope system achieves a noise floor of $0.37{}^{\circ}/\sqrt{\text{h}}/\sqrt{\text{Hz}}$ rate sensitivity of 0.32 mV/°/s and bias drift of 17°/h in [96].

Another dual-axis BAW gyroscope reported in 2010 is shown in Figure 13b. The gyroscope operates at 3.12 MHz in a near mode-matched condition (without tuning) and has a –1 dB bandwidth of ∼1.5 kHz. The device has a sensitivity of 15.0 μV/°/s using a 10 V DC polarization voltage within a linear full-scale range of 30,000°/s [97]. Another BAW gyroscope is reported from the same Lab above with a high frequency of 11 MHz using piezoelectric transduction [98]. As seen in Figure 13c, the silicon resonator is fabricated in a rectangular bar so that it can be considered as acoustic waveguides with finite dimensions. Different width to length ratios will have different resonance modes that can be approximately attributed to the Lamb modes of an infinitely long waveguide. Simulation and analysis prove that a prototype square gyroscope is optimized. The device is fabricated using a simple 4-mask process and the size is 300 μm × 300 μm × 20 μm. The orthogonal flexural resonance modes are used to provide energy exchange paths for the Coriolis-based resonant gyroscope in response to z-axis rotation. The gyroscope shows linear rate sensitivity of 20.38 μV/°/s. Figure 13d shows a 0.6 mm diameter, 20 μm thick BAW gyroscope presented by Nitzan *et al.* The gyroscope is fabricated using high-temperature, ultra-clean epitaxial polysilicon encapsulation, resulting in a good temperature sensitivity of −26 ppm/°C, a high Q of 50,000, high performance and small volume. The reported scale factor is 0.286 mV/(°/s), angle ARW is $0.006({}^{\circ}/\text{s})/\sqrt{\text{Hz}}$ and Allan deviation is 3.29°/h [99].

### 2.6. MESGs

**Figure 14.**
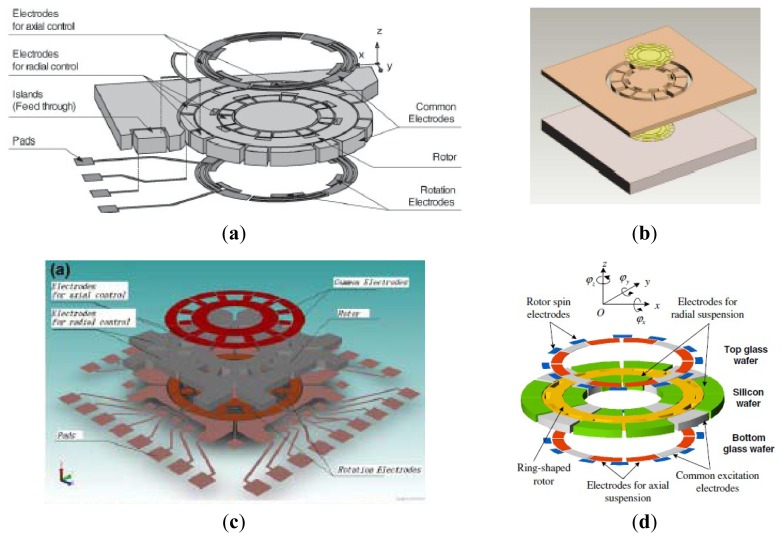
Various MESGs. (**a**) Tohoku University MESG. (**b**) University of Southampton MESG. (**c**) SJTU MESG. (**d**) THU MESG.

Conventional MVGs are sensitive to manufacturing tolerances. The MESG is developed to be insensitive to the tolerances. Due to the levitation of the rotor, the gyroscope can eliminate mechanical friction and obtain high precision. Murakoshi *et al.* first proposed the micromachined electrostatically levitated rotating gyroscopes shown in Figure 14a in 2003 [100]. This MESG consists of a triple glass-silicon-glass stack structure and stator electrodes that are symmetrically arranged around the ring-shaped rotor to form a capacitor for capacitive detection and electrostatic actuation. Damrongsak *et al.* from the University of Southampton also developed a MESG employing a levitated-disk proof mass, as shown in Figure 14b [101–103]. The gyroscope consists of a disk-shaped proof mass surrounded by suspension and spin electrodes. Suspension electrodes along the z direction and spin electrodes are located on the top and bottom of the disk. The disk is surrounded by electrodes for position control in the x and y-axis at its periphery. All the MESGs above can work as multi-axis inertial sensors.

In recent years, researchers from SJTU presented a novel MESG based on non-silicon MEMS technology, which can measure the dual-axis angular velocity and tri-axis linear acceleration, as shown in Figure 14c [104,105]. The MESG was fabricated based on LIGA or LIGA-like process. The rotor is suspended by electrostatic force through axial and radial electrodes and driven to rotate by the rotation electrodes. Suspension electrodes are located along the z-axis and rotation electrodes are located above and below the rotor. The rotor is surrounded by electrodes for position control in the x and *y*-axis at its circumference. Moreover, the position control for the suspension enables the gyroscope acting as a force-balanced tri-axis accelerometer. A rebalance loop controller must be used to improve the robustness of the MESG, such as PI controller [106], H_∞_ controller [107] and adaptive single neuron proportional integral (SNPI) controller. The sensitivity along axial direction is 1 V/g when it acts as an accelerometer. However, no detailed experimental results about the gyroscope sensitivity were reported.

Researchers from Tsinghua University (THU) proposed a MESG with a spinning ring-shaped rotor, as seen in Figure 14d [108]. The rotor is suspended by an electric bearing in five DoF and driven by a three-phase variable-capacitance motor. The electric bearing provides contactless suspension of the rotor, which allows the rotor to precess around two input axes that are orthogonal to the spin axis. Thus, the MESG can be used as a two-degree-of-freedom angular rate sensor by detecting the precession-induced torques. The prototype device is fabricated by bulk micromachining technique and rotates at a speed of 10,085 rpm at a high level of vacuum packaging. The experimental results of the rate gyroscope show that the input range is ±100°/s and the scale factor is 39.8 mV/°/s with a noise floor of $0.015{}^{\circ}/\text{s}/\sqrt{\text{Hz}}$ and a bias stability of 50.95°/h. Table 9 gives a summary of MESG gyroscopes mentioned above.

### 2.7. MSGs

**Figure 15.**
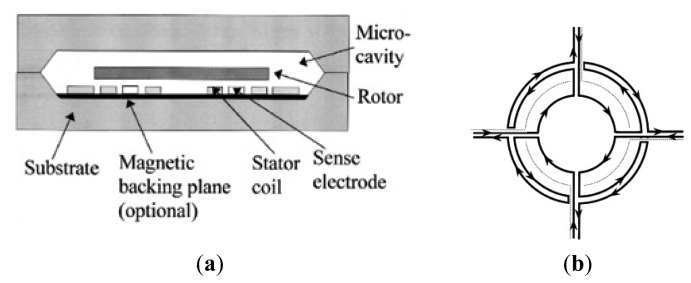
Schematic diagrams. (**a**) Cross-section view of a levitated micro-rotating gyroscope. (**b**) The four-phase micromotor.

The MESG needs a complicated feedback control circuit or a tuned circuit. To avoid the drawbacks mentioned above, diamagnetic levitation system with coils is put forward, owing to its advantages of simple structure, no energy input and no feedback control circuit. In 1997, Williams *et al.* from Nanyang Technological University (NTU) firstly proposed a levitated micromotor using in a novel rotating rate sensor [109,110]. Its structure includes five major parts, *i.e.*, top-shell, substrate, micro rotor, plane coil and sensing electrode, as shown in Figure 15a. The plane coil contains the suspended coil, the upside stability coil and the detection electric capacity electrodes, which are respectively used to produce the suspending force, the lateral stability force and detecting the rotor position. Figure 15b shows the four-phase plane coil structure schematic view. When a high-frequency current is input into the suspension coil, the procreant electromagnetic field will produce induction turbulent flow in the aluminum rotor, which will generate the electromagnetic repulsion to cause the rotor suspending. At the same time, superimpose the poly-phase current is superimposed in the coil to produce the rotational electromagnetic field to cause the rotor rotating at a high speed.

**Figure 16.**
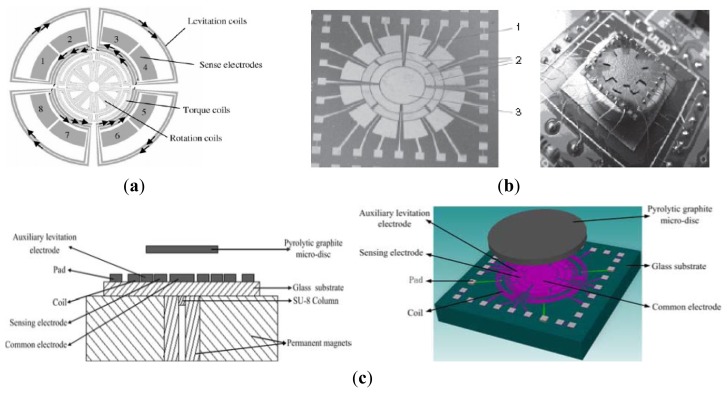
MSGs of SJTU. (**a**) The gyroscope designed in 2006. (**b**) The gyroscope designed in 2008. (**c**) The gyroscope designed in 2010.

**Figure 17.**
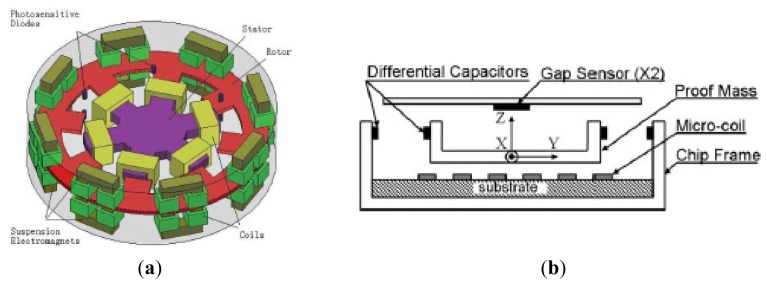
(**a**) University of Electronic Science and Technology suspended rotor gyroscope. (**b**) Schematic diagram of magnetic actuator.

Researchers from SJTU have proposed some other kinds of MSGs. Figure 16 shows views of the magnetic suspended gyroscopes with levitation rotor. In 2006, an electromagnetic micromotor with alumina rotor, which is stably levitated, rotated, sensed and controlled by independent coils and capacitance structure, as seen in Figure 16a, was presented in [111]. The key structure of the micromotor includes the levitation coils, rotation coils, torque coils, and sense electrode. The rotor is made of the pyrolytic graphite and metal electrodes, pads and SU-8 column post is fabricated by the MEMS process on the silicon substrate above the magnets. The rotation speed of 3,035 rpm is realized by a four-phase induction micromotor composed of the rotor and eight rotation planar coils carrying AC current. Figure 16b shows a micromotor whose rotor is levitated, rotated and constrained by the combination of static magnetic, electrostatic forces and torques reported in 2008 [112]. Unlike the driving method in [111], a speed rotor of over 10 rpm under atmospheric conditions is driven by a three-phase axial variable-capacitance motor with a 30 V driving voltage. The rotor is stably levitated to a height of about 0.70 mm over 200 μm of the magnets. Two year later, a new design, simulation, and fabrication, levitation experiment of an innovative micro-diamagnetic levitation system with coils were presented, as seen in Figure 16c [113]. The device consists of three main parts: micro-disc, stator and permanent magnet (PM). The pyrolytic graphite disc is levitated at the top of the stator. The front side of stator is made up of four coils, sensing electrodes, auxiliary levitation electrodes, common electrodes and pads. On the back side, SU-8 2100 resist is used to construct the column placement post for anchoring the PMs. The PMs are composed of two concentric ring-shaped magnets. The AC current is applied in coils to drive the rotor rotate at the high speed, which has advantage over the DC type in coils at the low speed. Recently, researchers from University of Electronic Science and Technology (UEST) of China studied one kind of LC tuning magnetically suspended rotor gyroscope [114]. The suspended rotor gyroscope is shown in Figure 17a. The gyroscope consists of suspension electromagnet, the rotor and the stator. The rotor is located in the center of the gyroscope. The suspension electromagnet consists of eight ferrite cores with coils, which are connected to the same numbers of capacitors. These suspension assemblies are right above and underneath the rotor. The 6-pole, 3-phase stator which has six coils on it is surrounded by the 8-pole rotor. When the rotor is displaced, the difference between the upper electromagnet voltage and the lower electromagnet voltage is measured. National Cheng Kung University (NCKU) of Taiwan presented a magnetic actuator design for single axis micro-gyroscopes [115].

Figure 17b shows a schematic diagram of the magnetic actuator. The magnetic actuator mainly consists of the micro-coils, proof mass, differential capacitors and gap sensors which are used to provide the feedback signal so that the magnetic force generated by the coil current can be controlled. However, when the magnetic actuator works as a gyroscope, it is really not economical and feasible to arrange other sensors, so the coil is not only an actuator to generate the attractive magnetic force, but also serves as the gap sensor. When the seismic proof mass can be controlled to oscillate in z-axis by tuning appropriate magnitude and phase angle of the applied voltage, then it would respond to move in y-axis as long as an angular excitation about x-axis is present. The differential capacitors are used to measure the displacement of proof mass in y-axis so that the angular rate about x-axis can be calculated. Table 10 shows a summary of MSG gyroscopes mentioned above.

### 2.8. Micro Fiber Optic Gyroscopes

The micro fiber optic gyroscope (MFOG) based on the Sagnac effect is now at a very advanced stage in aerospace guidance and navigation applications. The y have been used for at least two decades for a wide range of military and civilian applications. Optical gyroscopes have demonstrated high precision and widely dynamic ranges. Optical gyroscopes mainly include the ring laser gyroscope and the interferometer fiber optic gyroscope (IFOG). Compared with vibration gyroscopes, the FOGs have the advantages of no moving parts in the design, very high precision, long life time and robustness to the environment. However, there are limitations to their consumer applications because of the big size and very high cost of the RLG and IFOG designs. As MEMS technology improves, microoptical electromechanical system (MOEMS) gyroscopes combining MEMS technology with conventional optical technology appear to minimize the size and lower the cost.

**Figure 18.**
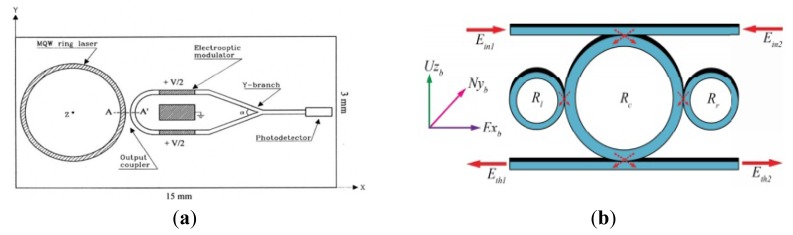
(**a**) Multiple quantum MFOG. (**b**) PANDA ring MFOG.

An accurate model and design of a fully integrated optical angular velocity sensor was firstly proposed by Armenise *et al.*, based on a multiple quantum-well microring laser, as seen in Figure 18a [116]. The gyroscope consists of an AlGaAs semiconductor ring laser (SRL) and some readout optoelectronic parts integrated on a single GaAs chip. The ring laser creates two counter propagating beams that are generated within the bidirectionally operating SRL. Due to the Sagnac effect, the rotation induced frequency shift, which is proportional to the angular rate, is detected by the system including a phase-shifter, a Y-junction and a photodetector. However, the performance is limited by lock-in phenomena due to backscattering. To avoid the lock-in phenomena, an indium phosphide (InP)-based angular rate sensor, which is not affected by lock-in has been recently reported in [117]. The readout system and the SRLs are integrated on the InP substrate. Actually, InP technology is one of the most attractive technologies for fully integrated optical gyroscopes. University of Southern California researchers demonstrated an all-buried InP–InGaAsP ring resonator which is laterally coupled to bus waveguides in 2004 [118]. The buried structure offers a guide to enhance optical coupling coefficients between the waveguides and reduce scattering loss caused by the resonator sidewall imperfections. Although scattering loss in InGaAsP/InP is almost six times larger than in silica-on-silicon due to the different index contrast sidewall roughness values, it can be minimized by optimizing the waveguide geometrical and physical parameters [119]. The guidelines to optimize the design of a velocity sensor based on an InP ring resonator were reported [120]. They minimize the propagation loss within the optical cavity down to 0.3 dB/cm to acquire a quality factor value of 1.5 × 10^6^. The optical power is 2 to 5 mW with a resolution of 10°/h and a bias less than 0.3°/h in 2013. Another 3-D axis MFOG based on InGaAsP/InP waveguides proposed by Sa-Ngiamsak *et al.* in 2012 is shown in Figure 18b. The gyroscope is comprised of a modified add/drop filter known as a PANDA ring resonator which consists of a single ring resonator with two lateral nano-ring resonators. The gyroscope can detect rotating angular velocity and horizontal velocity according to the different phase shift [121,122].

Despite the disadvantage of Rayleigh backscattering above, Micro-FOG accuracy is generally limited by the undesirable properties of Kerr, Faraday and thermal effects. Corning Incorporated presented air-core photonic-bandgap fibers (PBFs) that offer a radically new way for reducing the effects mentioned above [123]. Utilizing the air-core PBFs in a resonant fiber-optic gyroscope, a Stanford University group successfully proposed a resonant fiber-optic gyroscope (RFOG) to reduce Rayleigh backscattering, Kerr, Faraday and thermal effects. Compared with the traditional fiber which optical mode travels entirely through silica, the optical mode of the new gyroscope travels through air where all four effects are considerably weaker than in silica. They use a broadband source and quadrupolar winding to reduce deleterious effects. With a 235 m fiber coil, the minimum detectable rotation rate is 2.7°/h and the long-term stability is 2°/h [124]. Moreover, the thermal sensitivity is 6.5 times lower than that of the same gyroscope operated with a similar coil of conventional fiber [125]. Not until 2012 was the first public experimental RFOG using an air-core PBF as the sensing coil reported. The measured random walk is 0.055°/s, a long-term drift with a standard deviation is 0.5°/s and a peak-to-peak variation is 2.5°/s over 1 h [124]. The performance is further improved by using a laser to drive the FOG instead of a traditional broadband light with the noise and the bias drift of $0.005{}^{\circ}/\sqrt{\text{h}}$ and 1.1°/h, respectively [126].

**Figure 19.**
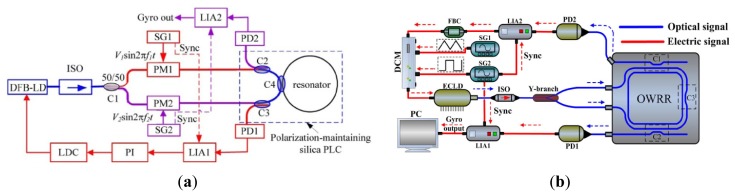
The circuits of RMOG. (**a**) Zhejiang University. (**b**) Beihang University.

Apart from the structure and material innovation, other signal process technologies are used to improve the performance. To reduce backscattering induced noise in a resonant micro optic gyroscope (RMOG), Ma *et al.* from Zhejiang University proposed a Carrier Suppression method [127]. As seen in Figure 19a, CW and counter CCW lightwaves are phase-modulated at different frequencies. Phase modulators (PMs) are driven by sinusoidal waveforms from signal generators (SGs) with different frequencies. The CW and the CCW lightwaves from the resonator are detected by the photodetectors (PDs). The output of the PD is fed back to the lock-in amplifier (LIA) and then controlled by a proportional and integration (PI) controller. Next, the laser diode controller (LDC) is used to cancel the fluctuations in resonant frequency and/or the central frequency of distributed feedback laser diode (DFB-LD). The rotation rate is detected through an open-loop readout system. Using the carrier suppression method, the gyroscope can reach a bias stability of 0.46°/s, which was the best one demonstrated in a silica waveguide ring resonator with a ring length as short as 7.9 cm till that time. The noise RMOG is further improved through double phase modulation using a FPGA-based digital signal processor. The equivalent input noise is as low as $3.752\text{nV}/\sqrt{\text{Hz}}$, which means the gyroscope can detect an equivalent Sagnac effect of 0.003°/s [128]. A current modulation technique used in an external cavity laser diode (ECLD) in Figure 19b was first proposed by a team at Beihang University. Test results show a bias stability of 2.7°/s (10 s integrated time) over 600 s, and dynamic range of 500°/s with a silica OWRR having a ring length of 12.8 cm [129].

### 2.9. Micro Atom Gyroscopes

Micro atom gyroscopes (MAGs) consist of nuclear magnetic resonance gyroscopes (NMRGs) and atom interferometry gyroscopes (AIGs) [130]. The principle of NMRGs is based on the Larmor precession while the principle of AIGs is based on the Sagnac effect. Compared with micromachined spinning or vibratory gyroscopes, NMRGs have the potential advantage that they contain no moving parts. Princeton University scientists proposed an atom spin gyroscope (ASG) based on an alkali-metal-noble-gas co-magnetometer in 2005. The gyroscope utilizes optically pumped alkali-metal vapor to polarize the noble-gas atoms and detect their gyroscopic precession. Spin precession due to magnetic fields as well as their gradients and transients can be cancelled in this arrangement. The rotation sensitivity is $5\times 10^{-7}\text{rad}/\text{s}\sqrt{\text{Hz}}$, which is equivalent to a magnetic field sensitivity of $2.5\text{fT}/\sqrt{\text{Hz}}$ by using a high-density alkali-metal vapor in a spin-exchange relaxation free regime [131].

**Figure 20.**
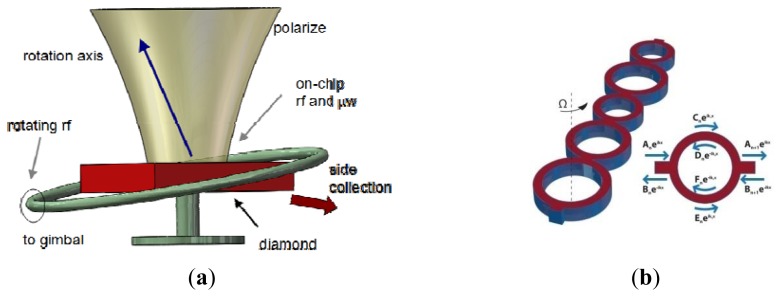
(**a**) MIT MAG. (**b**) Stevens Institute of Technology MAG.

Recently, MIT researchers presented a sensor that overcomes the limitations between long-time stability and high sensitivity by providing a sensitive and stable three-axis ASG in the solid state, as seen in Figure 20a. A high sensitivity is obtained by exploiting the long coherence time of the ^14^N nuclear spin. Long-time stability is improved by the coherent control of the quantum sensor. The reported sensitivity is $\eta ∼0.5(\text{mdeg}/\text{s})\sqrt{{\text{Hzmm}}^{3}}$ [132]. Atom interferometry gyroscopes have a potential sensitivity 10^10^ greater than optical gyroscopes, although they both detect inertial rotations via the Sagnac effect. In 2010, a Stevens Institute of Technology group presented an atom gyroscope with disordered arrays of quantum rings. The gyroscope consists of several rings for atom interference. The individual defect rings and the effects of disorder will lead to a more significant degradation of the phase sensitivity. Despite the large degradation in sensitivity, the gyroscope is still almost two orders of magnitude below the n^−1/2^ shot noise limit for 1% variations in velocity and ring size [133].

### 2.10. Micro Fluid Gyroscopes

**Figure 21.**
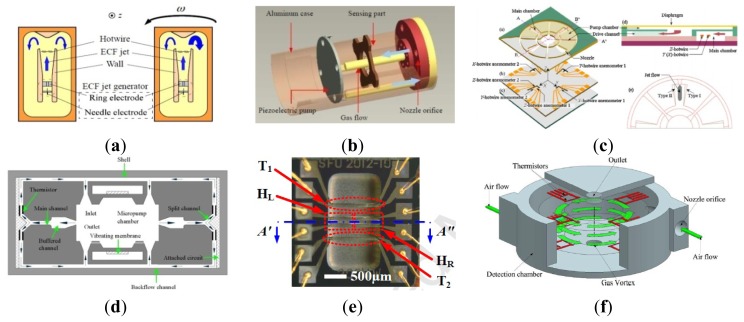
Various micro fluid gyroscopes. (**a**) Keio University gyroscope. (**b**) TSCL and RU gyroscope. (**c**) RU gyroscope. (**d**) HUST gyroscope. (**e**) Jamal Bahari gyroscope. (**f**) Northwestern Polytechnical University (NPU) gyroscope.

Among the consumer product applications, fiber optic or laser angular velocity sensors provide extremely precise information. However, the price is generally too high for wide use in the consumer field. Although vibratory angular velocity sensors are suitable for consumer applications because of their low cost and long life time, they are not robust enough for external impact/vibration sensitive uses because of the vibrating elements inside. Micro fluid gyroscopes (MFG) have been widely researched in Japan, at institutions such as the Tokyo Institute of Technology, Keio University, Ritsumeikan University (RU), Tamagawa Seiki Co., Ltd (TSCL), and New Technology Management Co., Ltd. In 2008, Yokota *et al.* first proposed the concept of a liquid rate gyroscope using an electro-conjugate fluid (ECF), as seen in Figure 21a [134]. This mode mainly consists of a jet generator (electrode pair), channel separation walls, hotwires and ECF inside. When the angular rate around the z-axis is applied to the model, the jet flow inside drifts due to the Coriolis force resulting in a difference in the electric resistance of two hotwires. As a result, the output voltage from the hotwire bridge will change because the electrical resistance of hotwires is related to temperature. This ECF liquid rate gyroscope is more sensitive to angular rate than gas rate gyroscopes. The volume of the liquid rate gyroscope is 40 × 60 × t7 mm^3^. The scale factor is −29 mV/(°/s) with an applied high voltage of 4.5 kV, which is 2.2 times more sensitive than the conventional gas rate gyroscope [135]. The hotwire bridge is also used in the semiconductor gas gyroscope proposed by Tamagawa Seiki Co., Ltd *etc.* This gyroscope, as shown in Figure 21b, consists of two main parts. One is a piezoelectric diaphragm pump, which is oscillated at a frequency of 7 kHz to create a continuous gas flow with a peak flow velocity. The other is the micro hotwires which are fabricated in lightly-doped p-type silicon with dimensions of 400 × 6 × 2 μm^3^ (length × width × thickness) [136]. In 2013, a MEMS-based dual-axis fluidic angular velocity sensor was presented by Ritsumeikan University (RU). The structure of the angular velocity sensor includes three layers: the top which consists of a pump chamber, drive channel, and main chamber, a hotwire anemometer to sense the angular velocity, and bottom layers respectively, as seen in Figure 21c [137].

The fabrication process of the gyroscope combines a standard MEMS process and a hot embossing process. It is actuated by a piezoelectric-actuated diaphragm through a valveless network channel and sensed by the hotwires. However, there are no details about the performance and cross-sensitivities. HUST in China reported a novel micro thermo-fluidic gyroscope utilizing dual directional liquids [138]. Compared with the traditional jet deflection method, the gyroscope consists of two pairs of symmetric microchannels splitting from the main channel. As seen in Figure 21d, when the Coriolis force is induced by external rotation, the fluid flow into the symmetric microchannels will be unequal, which results in different thermal convections between the fluid and thermistors inside the split microchannels. The angular rate is obtained by measuring the difference of the symmetric thermistors.

The MEMS thermal gyroscope proposed by Nanjing University of Science and Technology (NUST) and Simon Fraser University (SFU) is shown in Figure 21e. The MEMS thermal gyroscope consists of two symmetric heaters and two symmetric temperature sensors. Two heaters are alternately heated to create a bidirectional flow of expanding gas. By reducing the heaters' switching frequency, the linear acceleration effect of the thermal gyroscope can be efficiently compensated [139,140]. Chang *et al.* from NPU reported in 2013 a 6-DoF vortex inertial sensor that can detect three components of angular rate and linear acceleration. As seen in Figure 21f, the sensor is composed of two nozzle orifices, a cylindrical detection chamber with a diameter of 20 mm and a height of 7 mm and two outlets. The sensor uses a vortex gas flow instead of the traditional linear gas flow as the inertial mass to detect the angular rate and linear acceleration. The measured sensitivities of the gyroscope for the *x*-axis, y-axis, and z-axis are 0.429, 0.338, and 0.159 mV/°/s, respectively. The measured sensitivities of the accelerometer for the *x*-axis, *y*-axis, and z-axis are 0.185, 0.180, and 0.133 V/g, respectively [141]. Table 11 shows a summary of various micro fluid gyroscopes mentioned above.

### 2.11. Molecular Gyroscopes

**Figure 22.**
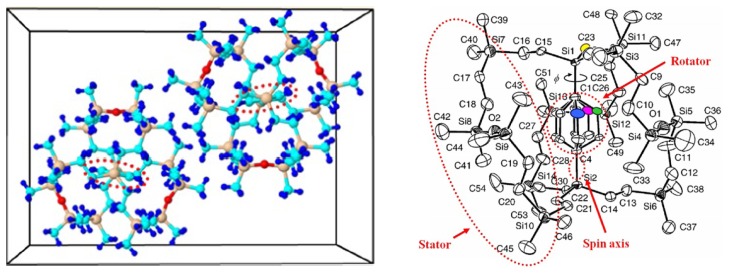
Molecular Gyroscopes.

Recently, molecular rotor systems have been emerging as promising candidates for functional nano-scale devices. As seen in Figure 22, a molecular gyroscope in a crystalline solid is particularly unique due to its variable physicochemical properties. Marahatta *et al.* in Japan theoretically analyzed the underlying mechanism of its rotational dynamics by utilizing the self-consistent-charge density-functional-based tight-binding (DFTB) method for crystal structures. They reported that DFTB semiquantitatively reproduced the unit cell molecular geometries of all three stable X-ray structures under the periodic boundary conditions. They found that the activation barrier for phenylene rotation was estimated to be about 1.2 kcal/mol. Compared with an open topology, the siloxaalkane frame in the crystalline molecular gyroscope under consideration effectively blocks strong intermolecular steric interactions experienced by the phenylene rotator. The dynamics simulation results based on the DFTB exemplified facile phenylene flipping show the remarkable ability of the DFTB method to predict the crystal structures and rotational dynamics of this type of crystalline molecular gyroscopes [142].

### 2.12. Special Micromachined Gyroscopes

#### 2.12.1. Slot-Structure Gyroscopes

**Figure 23.**
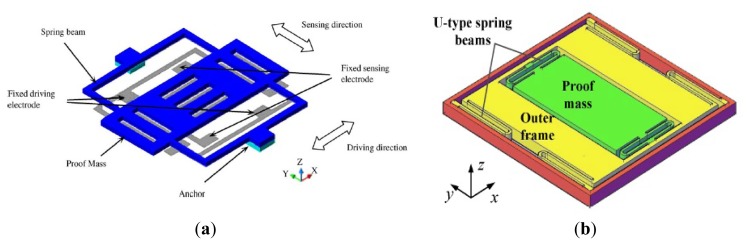
Slot-structure gyroscopes. (**a**) SIMIT. (**b**) Zhejiang University.

Xiong *et al.* from SIMIT presented a novel silicon micromachined gyroscope prototype with slots structure shown in Figure 23a [143]. This gyroscope, which is called a “slots gyroscope”, consists of a proof mass with slots linked up to a substrate by suspended springs and is fabricated by silicon–glass bonding and DRIE. Electrostatic driving and capacitive sensing are used in this gyroscope. The gyroscope can be operated at atmospheric pressure due to the high quality factors which are almost the same in the sensing and driving directions. The scale factor and non-linearity of the micromachined gyroscope are 20 mV/°/s and 0.56%, respectively, at atmospheric pressure.

Another interesting slot-structure MEMS gyroscope, seen in Figure 23b, was presented by a Zhejiang University team in 2013. The resonant frequency of this gyroscope can be tuned by novel triangular shape fixed electrodes under the proof-mass to overcome the disadvantage in the conventional slot-structure gyroscope that the resonant frequencies cannot be adjusted through variable area capacitors [144].

#### 2.12.2. Vibrating Beam Gyroscopes with High Shocking Resistance

**Figure 24.**
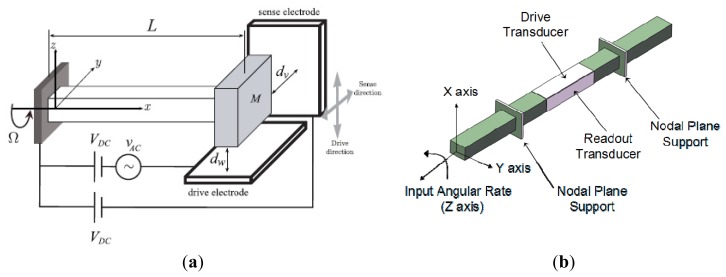
Vibrating beam gyroscopes. (**a**) Clemson University gyroscope. **(b**) Chongqing University of Posts and Telecommunications gyroscope.

A vibrating beam micromachined gyroscope is also a micromachined vibrating gyroscope making full use of the Coriolis force [145,146]. There are many advantages of this kind of gyroscope, such as low cost, small-volume and good shock resistance. A detailed mode and performance evaluation was performed by Esmaeili *et al.* in 2007 [145]. The governing equations are derived by using the Extended Hamilton's Principle with a general 6-DoF based motion. A similar rotating cantilever beam equipped with a proof mass at its end was proposed and analyzed by Ghommem *et al.* in 2010. As shown in Figure 24a, during operation, an alternating force oscillates the proof mass of the micromachined gyroscope. When this oscillating body (drive oscillations) is placed in a rotating frame, the Coriolis force produces secondary oscillations (sense oscillations), which are orthogonal to the driven oscillations. The angular velocity of the rotating frame can be estimated by analyzing the sense oscillations. This gyroscope is easily fabricated using micromachining processes. However, the measured electrical signal is complex because the proof mass is coupled with two electrodes in the drive and sense directions, which affects the precision of the gyroscope.

Another novel vibration beam structure gyroscope, named Node-Plane Supporting Vibration Beam Gyroscopes (NPSG), was presented by Chongqing University scientists in 2010. As seen in Figure 24b, the simple structure consists of a beam attached to foundation bed in the node point of beam. In order to actuate and sense the vibrations in the beam, the piezoelectric actuators are attached to the upper-surface and lower-surface, and the piezoelectric sensors are attached to the left-surface and right-surface. The reported results show that the shock resistance of the traditional Node Supporting gyroscope is below 2 × 10^2^ g, while the improved NPSG can reach 6 × 10^4^ g [147].

#### 2.12.3. 3D Spherical Shell Gyroscopes

**Figure 25.**
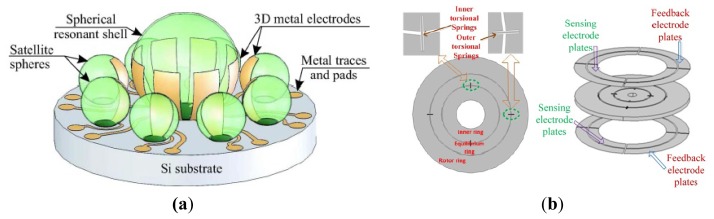
(**a**) 3D spherical shell resonator. (**b**) Southeast University MHG.

In order to utilize the high precision Hemispherical Resonator Gyroscope (HRG) MEMS scale, researchers from the University of California, Irvine first reported a 3D spherical shell resonator MEMS gyroscope fabricated by wafer-scale glassblowing [148,149], as seen in Figure 25a. It consists of a glass-blown spherical resonator shell and 3D capacitors formed by conductive metal films on 3D surfaces. The process starts by bonding a Pyrex glass wafer on a silicon substrate with pre-etched cavities. The flat metal electrodes are then patterned on the silicon-on-glass substrate. The wafer stack is then heated above 850 °C to induce plastic deformation of the metal-on-glass stack. Finally, the 3D spherical shells with integrated metal electrodes are available. A four-node wineglass mode could be utilized as the drive mode of a Coriolis vibratory gyroscope. The input rotation causes the transfer of energy from the drive mode to the sense mode. Sense mode vibrations are capacitively detected using 3D metal electrodes at a 45° angle. The 3D MEMS spherical shell gyroscope demonstrates a wide linear range of 1,000°/s.

#### 2.12.4. Microelectromechanical Hybrid Gyroscopes

Researchers from Southeast University (China) presented a new dual-axis micro-gyroscope called Microelectromechanical Hybrid Gyroscope (MHG) [150,151]. The MHG in Figure 25b consists of a rotor wafer (the left one) including the inner ring, equilibrium ring, rotor ring, torsional springs, and electrode plates with sensing electrode plates and feedback electrode plates. The rotor wafer is driven by a miniature motor to rotate rapidly. When an angular rate occurs along the x-axis or y-axis, the rotor wafer deflects, causing a change of capacitance values between the electrode plates and the rotor wafer. Thus, the input angular rate can be measured according to the corresponding change of capacitance values. To maintain the balance position of the rotor disc, an electrostatic feedback moment should be applied on the rotor wafer to rectify the rotor wafer deflection. A high bias voltage about 30 V is applied on the rotor wafer to achieve dynamical tuning due to the lack of the equilibrium ring negative stiffness. The scale factor is 1.42 mV/°/s and the scale factor non-linearity is 2.47% in full scale range of ±200°/s.

#### 2.12.5. Nano-Gyroscopes

**Figure 26.**
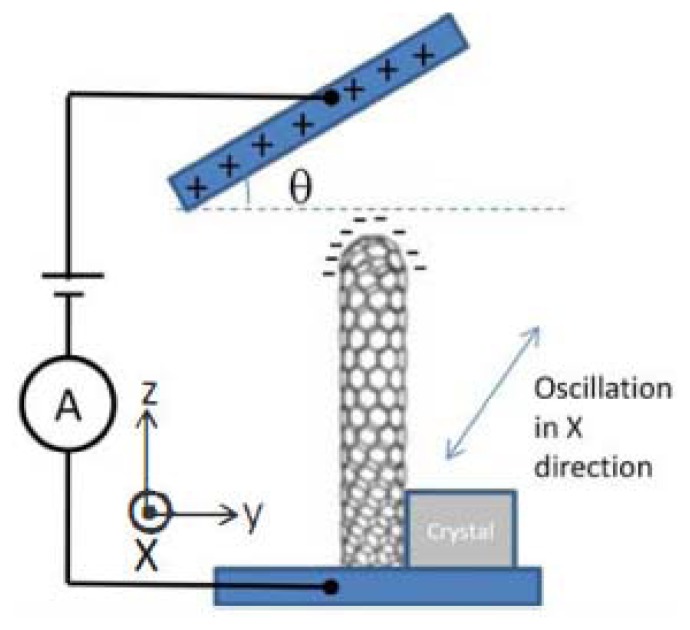
Nano-Gyroscope.

MVGs are now widely available at the commercial level and are starting to be available at the tactical level. However, it is hard for MVGs to reach strategy navigation level. Nanoscale gyroscopes with vibratory carbon nanotubes (CNT) have the advantages of low energy cost, high productivity, high resolution, large measurement scales and a potential total size of only several micrometers. Nagoya University presented a Carbon Nanotube (CNT) gyroscope based on the Coriolis effect [152]. As seen in the scheme in Figure 26, a CNT is vibrated in the x direction when there is a rotation around the z-axis applied to the y direction. The natural frequency of a MWCNT depends on the inner diameter, outer diameter and length. The CNT is driven by the electrostatic force to its natural frequency. A CNT with a length of 1 μm is used as emitter and an anode with a bias angle is etched by a Focused Ion Beam (FIB) which is set to the opposite to the CNT. An electrode is set near the CNT tip, which is employed to pulling the CNT for mechanical resonances by an AC voltage. The gyroscope can maintain good sensitivity up to 100 rad/s with a resonant frequency of 1 MHz and a mechanical quality factor of 10 k.

#### 2.12.6. Frequency Modulation Gyroscopes

**Figure 27.**
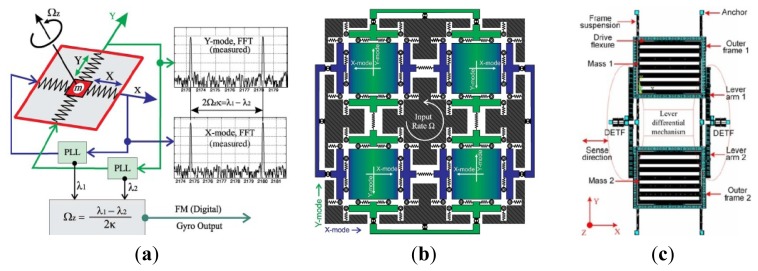
Frequency modulation gyroscopes. (**a**) Frequency modulation principle of UC Irvine. (**b**) Frequency modulation gyroscope of UC Irvine. (**c**) Frequency modulation gyroscope of Beihang University.

All MVGs based on Coriolis effect are amplitude modulation (AM) gyroscopes. In conventional gyroscopes, the input angular rate is amplitude-modulated by the drive mode velocity signal. They need high Q factors to improve the sensitivity, resulting in a constraint between Q factor and bandwidth. Moreover, AM sensors are also extremely sensitive to the value of the sense mode Q factor which will result in scale factor drifts caused by the ambient temperature and pressure. In order to solve the contradiction between the gain–bandwidth and dynamic range, Zotov *et al.* from UC Irvine first proposed an angular rate sensor based on mechanical frequency modulation (FM) of the input rotation rate [153,154]. The schematic of the closed-loop operated gyroscope based on the mechanical FM is shown in Figure 27a. The sensor consists of a symmetric, ultra-high Q, silicon micromachined Quadruple Mass Gyroscope (QMG) and a new quasi-digital signal processing scheme which takes advantage of a mechanical FM effect. The input angular rate is only proportional to the frequency split (*λ*_1_− *λ*_2_) The constant coefficient κ depends on the gyroscope design. The mechanical structure of the QMG mechanical sensor is shown in Figure 27b. The gyroscope comprises four identical symmetrically decoupled tines with linear coupling flexures as well as a pair of anti-phase synchronization lever mechanisms for both the x- and the y-modes. The complete x-y symmetrical structure improves robustness against the fabrication imperfections and frequency drifts. The test shows a scale factor of 2.367 mHz/(°/s) and nonlinearity of less than 0.2% in wide range of input range up to 18,000°/s. Allan variance of the FM sensor shows an ARW of $1.6{}^{\circ}/\sqrt{\text{h}}$, bias instability of 27°/h, and a dynamic range of 128 dB in the FM regime of operation (from 50°/h to 18,000°/s).

Figure 27c shows a double-ended tuning fork (DETF) gyroscope proposed by Li from Beihang University, which utilizes resonant sensing as the basis for Coriolis force detection instead of displacement sensing. The device is fabricated by the silicon on glass (SOG) micro fabrication technology. The gyroscope consists of two proof masses, a pair of DETF resonators and two pairs of lever differential mechanisms. The lever differential mechanism is responsible for the transmission of the differential Coriolis forces into one common force acting in the longitudinal direction of the DETF. When the two masses move toward each other or away from each other, the opposite Coriolis forces from the two masses are transferred to one common force. The common mode acceleration error is cancelled because the transferred force is differential. The rotation rate applied to the device can be estimated by demodulating the DETF resonant frequency and detecting the resonant frequency difference. The gyroscope has a frequency sensitivity of 12.535 Hz/°/s and a mechanical noise floor of $7.957{}^{\circ}/\text{h}/\sqrt{\text{Hz}}$in air [155].

## 3. Micromachined Gyroscope Circuitry

As another necessary part of the whole micromachined gyroscope system, micromachined gyroscope circuitry research is increasing around the World. Most of the micromachined gyroscope circuitry can be divided into two functions, one of which is called drive oscillation or primary oscillation for maintaining the gyroscope vibrating along the driving direction, and the other is called sense oscillation or secondary oscillation for angle rate detection. In order to maintain the primary oscillation, various driving circuits are used such as automatic gain control (AGC) to control the amplitude of the driving signal, phase locked loops (PLL) to control the phase of the driving signal and other control circuits to improve the performance of micromachined gyroscopes in the closed-loop drive circuit. The open-loop or closed-loop sensing circuits are applied to the gyroscope system for angle rate detection. Moreover, the defects caused by structure design and fabrication, and the influence of the application environment can be improved by the appropriate circuits. To satisfy the requirement of high precision and high stability, various electronic technologies are adopted in the gyroscope circuitry including temperature compensation, scale factor compensation, quadrature compensation, mode matching and so on. Different gyroscope structures require different circuits. Here, we are mainly concentrated on some typical analog and digital circuits, and some special circuits.

### 3.1. Typical Analog Circuitry

#### 3.1.1. Analog AGC in Drive Mode

**Figure 28.**
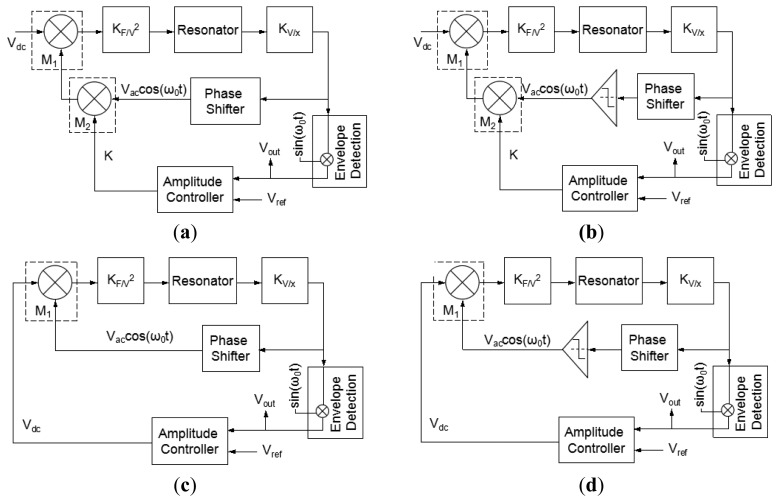
Different kinds of AGCs. (**a**) AC sine wave control. (**b**) AC square wave control. (**c**) DC sine wave control. (**d**) DC square wave control.

In order to maintain the micromachined gyroscope vibrating at its resonant frequency, typical driving circuits including AGC or PLL are introduced. AGC can be achieved by controlling either the DC or AC component of the excitation signal, while the AC signal can be either a sine or square wave. The different technologies have been described in [156], as seen in Figure 28.

Figure 28a,b are the AC amplitude control loops. The phase shifter (PS) is used to match the resonant frequency, and the signal after amplitude detection is used to control the AC amplitude of the excitation signal in the amplitude controller. M_2_ is a multiplier used as a variable-gain amplifier (VGA) to control the amplitude of AC signal. M1 is the multiplier to multiply the AC and DC signals as the whole excitation signal. Figure 28c,d are the DC control loops. Unlike AC control, the DC amplitude is controlled by the amplitude controller directly and there is no multiplier M_2_. The comparator is used to make the amplitude constant and the excitation signal into a fixed amplitude square wave.

**Figure 29.**
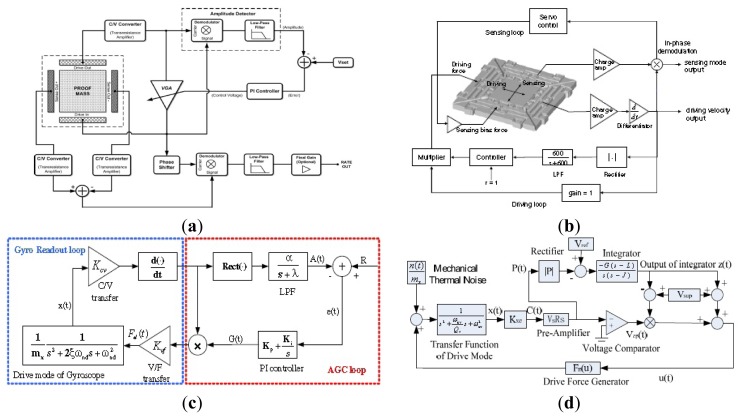
Analog AGC schematics. (**a**) Middle East Technical University. (**b**) Seoul National University. (**c**) PKU. (**d**) Southeast University.

An analog driving circuit like the schematic in Figure 28a is applied in [39], as seen in Figure 29a. The AGC closed-loop system is used to generate and control the stable drive mode oscillations. The preamplifier stage is a capacitance-to-voltage converter. VGA is used to satisfy the phase requirements for starting self-oscillations along the drive mode. The amplitude is detected by amplitude detector including demodulator and low pass filter, and then controlled to a desired amplitude signal (Vset) by using a proportional–integral (PI) controller which can minimize the error between the reference value and detection value. The output of the PI controller can continuously adjust the gain of VGA to keep the AC amplitude of the excitation signal constant at the desired level.

Figure 29b shows an AGC loop designed by Seoul National University workers. The AGC loop consists of a charge amplifier, differentiator, envelope detection part using a rectifier and low pass filter, controller, and multiplier [157]. In [158], the AGC with PI controller was presented to make the gyroscope achieve a constant amplitude vibration at its resonant frequency. Figure 29c shows the AGC loop with PI controller. In [157], the Lyapunov criterion is utilized in the amplitude controller to achieve the AGC loop stable, while a PI controller is adopted in the amplitude control in [158] to realize the self-oscillation of the gyroscope. Their amplitudes are both detected by the rectifier and low-pass filter (LPF). The principle of PI controller is similar to that in Figure 29a. The DC signal out of PI controller is multiplied by the signal out of the differentiator that is used to transform the displacement signal into a velocity signal.

Xia *et al.* from Southeast University also proposed an AGC driving loop in [159]. Different from the AGC in [158], the DC amplitude is controlled by a PID controller rather than the PI controller to get better response times. In [160,161], a new closed-loop drive scheme was presented which can decouple the phase and the gain of the closed-loop driving system, as seen in Figure 29d. The amplitude is controlled by the branch circuit above, and the phase is controlled by the branch circuit below. The “voltage comparator” is the key component of the closed-loop driving to output an invariable amplitude and only to reserve the phase information, so the phase conditions of the closed-loop are separated from the amplitude conditions. The circuit is beneficial for parameter adjustment and optimization because the amplitude and phase control are fully decoupled.

#### 3.1.2. Other Analog Driving Circuitry

**Figure 30.**
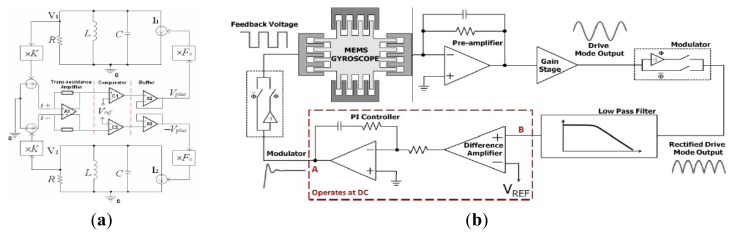
(**a**) Harbin Institute of Technology driving circuitry. (**b**) Middle East Technical University driving circuitry.

Most of the closed-loop driving circuits contain an AGC module in order to control the amplitude of the output signal. Other driving circuits [162,163], as seen in Figure 30, are also adopted in gyroscope systems to offset the influence of the AGC circuitry on the gyroscope sensitivity and stability. In Figure 30a, the key component of the closed-loop system is the comparator instead of the AGC circuit to control the amplitude of the driving AC voltage. Compared to the AGC circuit, the comparator is operated in the nonlinear range, so it would not restrict the linear range of the whole circuit. No matter what amplitude the input signal is, the output signal of the comparator is a square wave with constant frequency which is not related to the input amplitude. The technology is simple and easily achieved in ASIC. However, the Q value must be high when the comparator is used. Another driving circuit is shown in Figure 30b. The amplitude of the excitation signal is controlled by a PI controller and the frequency is set at a constant value through the switches. PI control has the advantage of zero steady-state errors, but this driving circuit will create a trade-off between the stability and settling time because of the pole of low frequency. To solve the contradiction between the stability time and setting time, a pole-zero cancellation method is used in [163].

#### 3.1.3. Analog Sensing Circuitry

**Figure 31.**
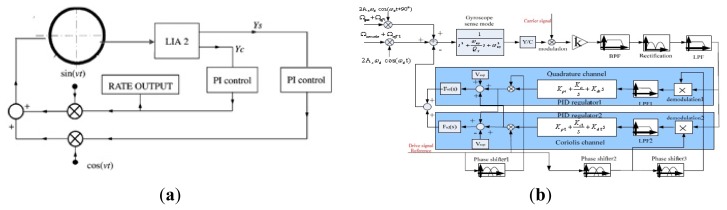
(**a**) University of South Carolina force-to-rebalance control circuitry. (**b**) Southeast University force-to-rebalance control circuitry.

The analog sensing circuitry has two categories: open-loop and closed-loop. Open-loop detection with its advantages of easy realization, reduced additional noise and high efficiency has been widely used in [39,164,165]. Typical open-loop sensing circuitry is shown in Figure 29a. Different sense outputs are picked up by preamplifier stages, next converted to a single-ended signal, then demodulated by using phase-sensitive demodulation with a carrier signal generated from the primary oscillation, and finally filtered by a LPF to provide a low frequency output that is proportional to the applied angular rate input. There will be two demodulators if the sense output is modulated by the high frequency signal. Due to a variety of advantages over open-loop such as noise elimination, better stability, mode matching and compensation, all kinds of closed-loops are adopted in gyroscope sensing circuits. A typical closed-loop sensing circuitry called force-to-rebalance is the main focus in [166]. The complicated closed-loop sensing circuitry will be discussed in the other section. The force-to-rebalance sensing circuitry is similar to the driving circuitry to generate a force to cancel the Coriolis force applied on the secondary vibration mode. A single-channel closed-loop control used in sense mode cannot completely counteract the useful signals, the quadrature signal and even the offset error, because this single force feedback is phase insensitive before performing synchronous demodulation to Coriolis plus offset and quadrature plus offset components. Thus, a dual-channel closed-loop control (quadrature part and Coriolis part) is used in the sensing circuit in [159,166,167], as seen in Figure 31.

In Figure 31a, the output signal can be demodulated by a lock-in amplifier (LIA) to produce two parts which are called Coriolis signal and quadrature signal. The PI controller works as a compensator to improve the system bandwidth and stability. A more detailed schematic diagram is shown in Figure 31b. The dual-channel force-to-rebalance control circuitry mainly consists of a Y/C module (the conversion coefficient from displacement in the sense direction to variable capacitor), filters, rectification module (for first demodulation of both signals), multipliers, PID compensators and phase shifters. The quadrature channel and Coriolis channel are separated by the second demodulation with the phase difference of 90°. The PID is used to improve the system bandwidth and stability. Different from the closed-loop in Figure 29a, the preloaded voltage has the advantage of adjusting the electrostatic force feedback generator Fn(s). The similar process is used to force rebalance control in the sense mode in [167]. However, different from the PID regulator in [159], a lead-lag compensator plus a first-order LPF is adopted as the controller. Moreover, a notch filter is used to compensate the peak located in the difference of these two frequencies and a limiter is used to obtain the control voltage to avoid the saturation of the electronic devices.

### 3.2. Typical Digital Circuitry

The analog circuitry has the defects of additional noise, temperature drift, and difficulty for self-testing, self-calibration, or other intelligent functions. Thus, digital circuitry is widely used to solve the problems mentioned above. Unlike analog circuitry where driving and sensing circuitry are achieved on separate devices, both digital driving and sensing circuits are placed on the same chip. Therefore, we will discuss the driving and sensing circuitry simultaneously here. The digital driving circuits, most of which include AGC, PLL and AGC + PLL, have been used in digital driving systems based on a digital signal processor (DSP) or a field programmable gate array (FPGA). AGC is easily achieved in either analog or digital systems. The continuous time AGC for analog systems is analyzed in the analog part and the discrete time AGC for the digital system is analyzed in [168]. However, the PLL module is difficult to achieve in the analog system, while it is easily achieved in a digital system. In [169,170], the PLL is used in the gyroscope system to track the natural frequency of a vibrating resonator. The principle of PLL is presented, and its stability and resolution are analyzed when used in a gyroscope system. Similar to the analog circuitry, the digital sensing circuit can also be divided into two categories: open-loop and closed-loop, which can be achieved in the DSP or FPGA.

**Figure 32.**
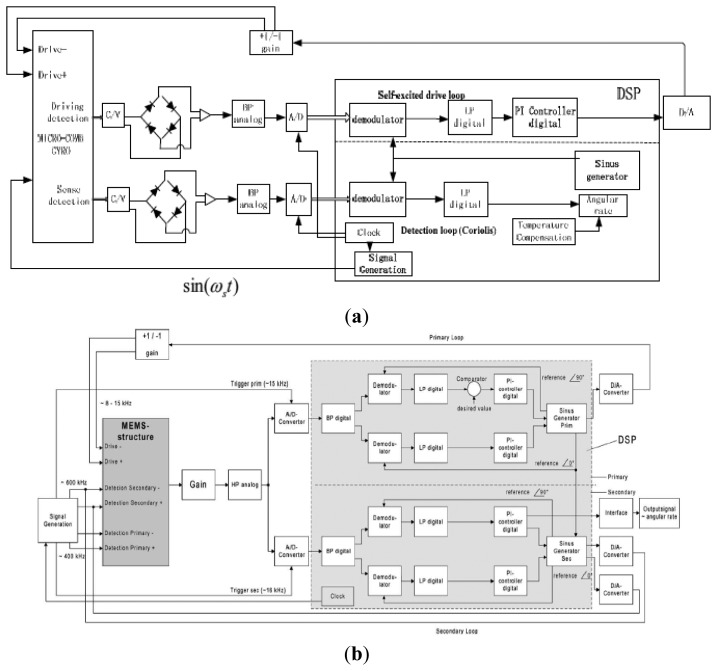
DSP circuits of (**a**) PKU. (**b**) HSG-IMIT.

In [171–174], some novel digital systems based on DSP are presented. The digital circuitry based on DSP of PKU is shown in Figure 32a [171]. The main functional modules of this loop include: C/V conversion, diode peak detector ring, A/D convertor, demodulator, PI controller and D/A converter. The diode ring peak detector can work as a demodulator to get the harmonic vibration signals reflecting the actual status of the gyro vibration. Demodulation and AGC control are achieved in the DSP. In [172], a digital readout system is presented to detect small capacitive signals of a micromachined angular rate sensor. The scale-factor was measured through the deflections of the movable masses under gravity. A calibration software algorithm and PLL are achieved in the DSP to calculate the scale factor and to maintain the primary oscillation, respectively. Figure 32b shows the new digital readout electronics for capacitive measurement [173]. The driving part consists of a phase and an amplitude control loop. They are all realized with a PI controller to accommodate the amplitude and frequency changes due to the variation of the resonant frequency and Q value with temperature. In [174], the multi-channel analog to digital interface is optimized for detection of small electrical signals and the digital to analog interface circuit produces a wide range of the actuation and detection voltage in gyroscopes.

For the open-loop sensing circuitry whose principle is similar to the analog circuit in [158], the analog front end is same as that in driving circuitry. To separate in-phase and quadrature signal, a demodulation algorithm of the least mean square demodulation (LMSD) is used in DSP. The angular rate is obtained after the digital low pass filter. For the typical closed-loop detection circuitry, compared digital circuitry in Figure 32b and analog circuit in Figure 31b, the process of the gyroscope signal is similar. There are also two channels: one is the quadrature channel and the other is Coriolis channel, which are separated by the digital demodulation with the phase difference of 90°. A PI controller is used to improve the system bandwidth and stability. The signal after the PI controller is directly proportional to the angular rate.

**Figure 33.**
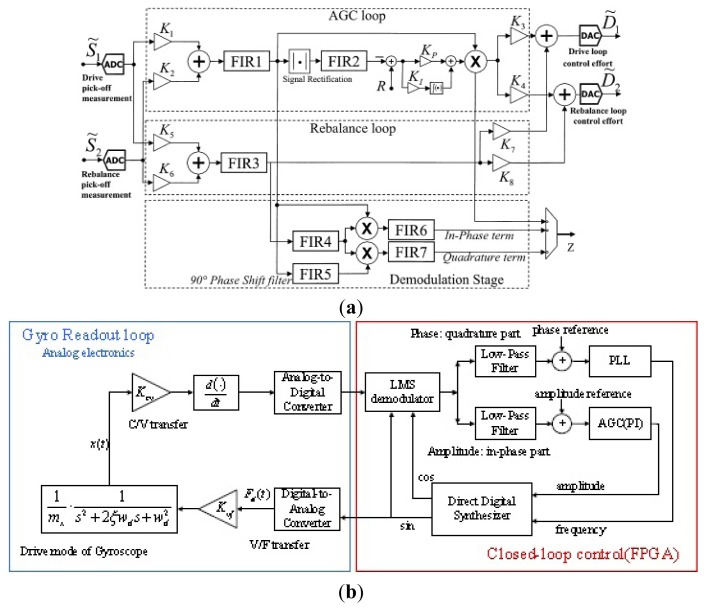
FPGA circuits. (**a**) JPL. (**b**) PKU.

Compared with the DSP, FPGA is a high performance device integrated with millions of digital logic elements, which can perform more complex numerical computing, logic decision and measuring-control functions, even with low power consumption and fast parallel processing. Two typical driving systems based on FPGA are shown in Figure 33: the JPL system and Peking system. JPL has successfully developed a hardware platform for integrated tuning and closed-loop operation of MEMS gyroscopes. The control of this device is implemented through the digital design on a FPGA, as shown in Figure 33a [175,176]. The AGC block in the driving circuitry consists of FIR1, the rectifier, FIR2, the proportional-integral (PI) compensation and programmable limiters on the integrator and AGC gain. The amplitude is detected through a rectifier and a low-pass filter. The PI compensation works as a comparator to generate the amplitude error signal and some compensation. The amplitude after the PI controller is stable when its error is minimum. For the rebalance loop detection circuitry, FIR4 and FIR5 are typically used to shift signal phases for the phase sensitive demodulation. FIR6 and FIR7 are low-pass filters located after the multipliers. The output of FIR3 is demodulated into in-phase and quadrature signal. The in-phase signal is directly proportional to the input angular rate. FIR3 is used to generate a compensation for the rebalance loop.

No PLL is adopted to control the resonant frequency in the digital JPL driving circuitry. In order to track the resonant frequency stably, the PLL based on FPGA is used in [177,178]. In [177], as seen in Figure 33b, the amplitude is kept constant by the AGC method with a PI controller while the phase is controlled by phase locked loop (PLL). The displacement of the gyroscope is demodulated by the adaptive filter-least mean square (LMS) method, where the in-phase part and quadrature part are separated to control the amplitude and the phase respectively. The PLL module mainly consists of a phase detector and a controlled oscillator realized by a direct digital frequency synthesizer (DDS). However, DDS will consume a lot of hardware resources in FPGA due to its non-flexible look-up table method. Coordinate Rotate Digital Computer (CORDIC) algorithm has been implemented in fiber optic gyroscopes to lock the laser resonant frequency [179]. In [178], the numerical control oscillator (NCO) is realized by CORDIC algorithm. To demodulate into the in-phase and quadrature signal, a better adaptive varying step LMSD approach can be realized to minimize the mean square error between the input signal and the output signal. Compared with the constant step LMSD, the adaptive varying step has the advantages of high-speed and low-noise.

### 3.3. Special Circuitry

#### 3.3.1. Sigma Delta

In order to realize the digitalization of micromachined gyroscopes, an analog to digital converter (ADC) is needed. Compared with traditional ADC, such as dual slope ADC, successive approximation ADC, parallel comparison ADC *etc.*, sigma-delta ADC has the advantages of low power consumption, high precision, high flexibility and ease to achieve in ASIC [180]. On the other hand, in order to improve the performance in terms of resolution and lower sensitivity against temperature and process variations, the open-loop readout cannot satisfy the future markets demand. The temperature influence on the quality factor and the process imperfections in the mechanical structures will result in the error component which may exceed the expected measured signal. For the primary oscillation of a gyroscope, these problems are solved by a drive loop which consists of the PLL and AGC modules. For the secondary mode, it is beneficial to improve a closed-loop as much as possible in the digital domain. Therefore, the readout based on the force feedback mechanism is widely used in the gyroscope system. An advantage of force feedback to the secondary mode is that the dynamic range of the readout can be significantly improved.

**Figure 34.**
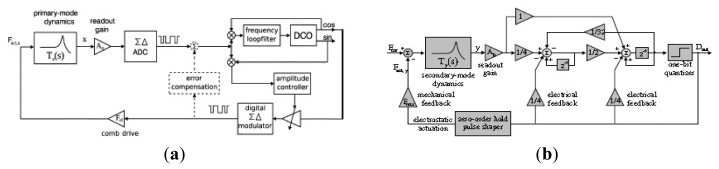
Ghent University of Belgium ΣΔ circuits. (**a**) drive mode. (**b**) detection mode.

With so many advantages of ΣΔ force feedback, Raman *et al.* from Ghent University of Belgium proposed the interface circuitry based on electromechanical ΣΔ technology for both primary and secondary modes [181–183], as seen in Figure 34. For the primary oscillation in Figure 34a, the x displacement is measured by continuous-time (CT) readout circuit and converted to the digital domain by a conventional switched-capacitor ADC. The phase shifter and amplitude controller are realized in the digital domain. The driving force is obtained from the cosine signal, generated by the DCO. Then the multi-bit digital signal is converted into a one-bit signal with a digital ΣΔ modulator and further used for actuation. In the driving circuitry of primary mode, the error compensation is added to the frequency tracking loop to compensate the parasitic electrical coupling. For the secondary mode, as seen in Figure 34b, a one-bit force-feedback for the readout is used for digital readout. However, there will be more quantization noise in the ΣΔ force feedback loop. As we know, the quantization noise should be below the electronic noise of the readout front-end in the working frequency range. Therefore, the noise transfer function (NTF), which transfers quantization noise to the output of the force-feedback loop, should be designed to minimize the quantization noise.

In [182], an optimal unconstrained architecture for such force-feedback loop was presented. Considering the mixed electrical and mechanical systems, an electrical resonator which is built by applying local feedback to a delay and a non-delay integrator is added to the loop to provide a notch in the NTF at the operating frequency of the gyroscope.

**Figure 35.**
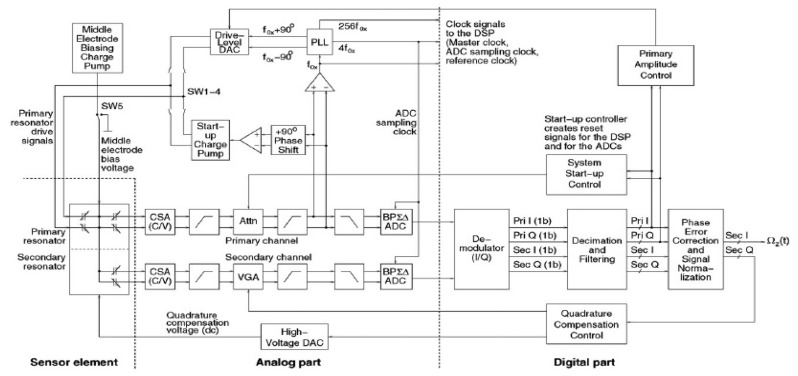
Gyroscope circuitry of Helsinki University of Technology.

Figure 35 shows an interface and control electronics designed and implemented by Helsinki University of Technology researchers for a bulk micromachined capacitive gyroscope with the electrical band-pass sigma delta A/D Converter [184,185]. The system is composed of the ASIC that implements the analog parts and a FPGA chip that implements the digital signal processing (DSP) part. The capacitive signals are firstly converted to voltage with charge-sensitive amplifiers (CSAs). After capacitance-to-voltage conversion, the signals are filtered and amplified, which means their levels are normalized with a tunable attenuator in the primary channel and a variable gain amplifier (VGA) in the secondary channel. Next, the signals are converted into the digital domain with bandpass ΣΔ ADCs. The outputs of both ADCs are synchronously demodulated to in-phase (I) and quadrature (Q) components in the DSP. Then, they are filtered and digitalized to achieve the final desired accuracy. Finally, the phase error correction is performed. Thus, the Coriolis signal is normalized by dividing the primary signal amplitude as the angular rate output and the quadrature signal for compensation control the clock of system and phase shift is generated by a comparator which converts the sinusoidal primary signal into a square wave and a PLL which is locked to the comparator output. The driving amplitude is controlled in the DSP. The mechanical quadrature signal is compensated using a feedback dc voltage which is generated by a 7-bit high-voltage (HV) DAC (digital to analog converter). A quadrature compensation controller sets the HV DAC output voltage to make the quadrature error zero and the gain of the VGA to maximize the dynamic range of the secondary channel. The output signal of the gyroscope has a very narrow bandwidth centered at the resonant frequency of the excited resonator. Therefore, a band-pass ΔΣ A/D converter is appropriate. Regardless to the mechanical model, a double-delay (DD) resonator is used in the ΣΔ band-pass ADCs for the accurate resonant frequency despite the capacitor mismatch.

**Figure 36.**
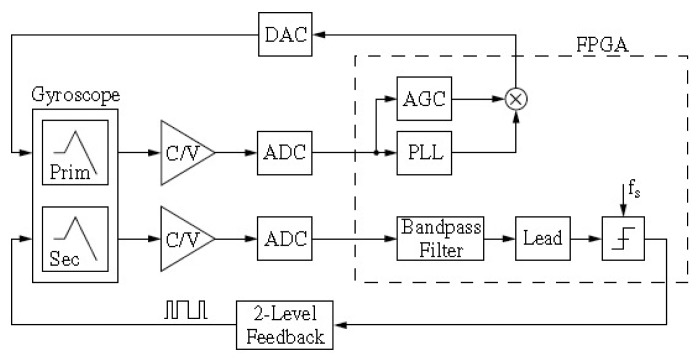
HSG-IMIT ΣΔ modulation circuitry.

Northemann *et al.* from HSG-IMIT demonstrated a MEMS gyroscope system with extensive use of ΣΔ modulation in both primary and secondary modes [186,187], as seen in Figure 36. There is a band-pass digital-to-analog converter (DAC) instead of discrete DAC driving the primary mass into resonance using a two-level driver. This technology can reduce the analog circuit complexity enormously. As seen in the driving loop, the circuitry mainly consists of the C/V, AGC, PLL, quantizer, and the fourth-order band-pass ΣΔ DAC. The 12-bit input signal of the DAC is a square wave signal with resonant frequency provided by the PLL and amplitude controlled by the AGC. Then, the signal converted to a one-bit signal with a fourth-order band-pass ΣΔ DAC implemented on the FPGA. The one-bit signal is applied on the primary mass for actuation using a two-level driving stage. Compared with the multi-bit ΣΔ DACs, the one-bit ΣΔ DACs can reduce the circuit complexity significantly but the quality factors must be large enough. For the detection loop, the signal is detected by a CT C/V converter. Its output is converted into the digital domain by an ADC. The second-order CT band-pass filter and the lead compensator are implemented on the FPGA for emulating CT behavior and ensuring the highest flexibility. Then, the one-bit quantizer is used to convert the signal into a bit stream. The digital output signal of the ΣΔ modulator is used to generate electrostatic force feedback by applying two-level voltages on the separated feedback electrodes for compensating approximately the deflection of the proof mass.

**Figure 37.**
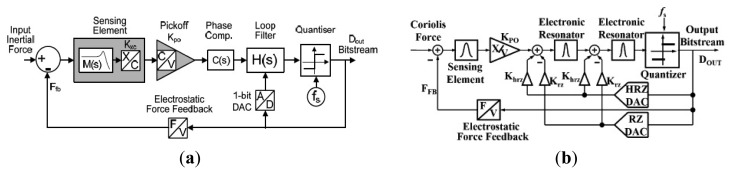
University of Southampton ΣΔ modulation circuitry. (**a**) The schematic of EMΣΔM. (**b**) The band-pass 6th order ΣΔ loop.

Figure 37 presents a novel high order continuous-time, force-feedback band-pass electro-mechanical sigma delta modulator (EMΣΔM) control system for the detection mode of micromachined vibratory gyroscopes [188–190]. Stability and performance are mainly dependent on the chosen architecture and the choice of the various gains in the pick-off circuitry and signal paths. The generic schematic of EMΣΔM is shown in Figure 37a. It consists of the micromachined sensing element, the pick-off circuit that converts capacitance to a voltage, a phase compensator (which may not be required if the sensing element is over-damped), an electronic loop filter consisted of several integrators and minor feedback or feedforward loops, a clocked one bit quantizer and a feedback block. The feedback block converts the feedback voltage into an electrostatic force applied on the proof mass and to rebalance the inertial force.

Figure 37b shows the band-pass 6th order ΣΔ loop for the detection mode as an example. The sensing element acts as a mechanical resonator. The gain K_PO_ represents the conversion gain of displacement to a voltage. Two electronic resonators are cascaded to provide additional noise shaping which serves as a phase compensator in the band-pass sigma-delta modulator. The multi-feedback topology should be adopted because excess loop delay in continuous-time ΣΔ modulators through a half-return-zero (HRZ) DAC and a return-zero (RZ) DAC. The different tunable gains K_HRZ_ and K_RZ_ are used to provide multi-feedback waveforms to maintain the same frequency response. A one-bit quantizer is used to output the bit stream and control the HRZ and RZ DACs, and also control the conversion from voltage to electrostatic feedback force.

#### 3.3.2. Mode Matching

**Figure 38.**
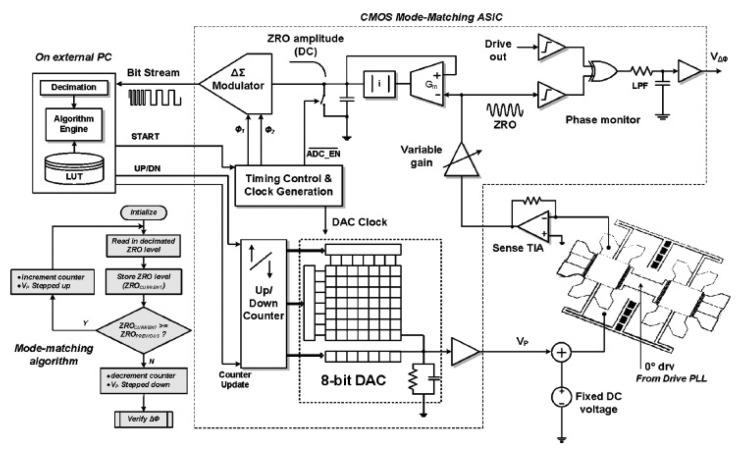
Georgia Tech mode matching system.

In order to improve the sensitivity, it is necessary to increase the energy transfer between the primary and secondary modes, which can be achieved by ensuring that the vibration frequency of the primary mode is in the bandwidth of the secondary mode. Moreover, the sensitivity is maximum when the resonant frequencies of the two modes are matched. However, perfect frequency matching is rather unfeasible only relying on fabrication because of inherent tolerances and defects associated with the manufacturing process. On the other hand, the parameter is sensitive to the temperature changes causing additional frequency mismatch. Thus, electronic technology is effectively implemented in the gyroscope to improve the performance robustness against mismatches. In order to match the resonant frequency of the two modes, a method of electrostatic force feedback in the secondary mode system was proposed by Georgia Tech in [13,25], as seen in Figure 38. Mode matching is achieved by increasing the DC voltage (Vp) on the MEMS structure until electrostatic spring softening to decrease the sense frequency until equal to the drive frequency. The mode-matching condition is attained by maximizing the amplitude of residual ZRO in the secondary mode. Additionally, under mode-matched conditions, there is a distinct 90° phase shift between the drive output and ZRO. The automatic mode-matching algorithm involves an iterative increment of V_P_ until the residual ZRO amplitude becomes maximum. Further, the 90° phase difference is monitored to ensure that mode matching has indeed been achieved. Moreover, once the mode is matched, the sensor bandwidth can be controlled by varying Vp to separate the drive and sense resonant frequencies. Another method for mode matching is implemented in [191,192], where it is achieved by adjusting (like PLL) the 90° out-of-phase relationship between drive frequency and sense frequency. The control voltage of mode-matching loop changes the phase of output signal. When the phase is locked, the exact control voltage will force the sense frequency to become equal to the drive frequency. However, both methods above require zero or constant angular rate inputs. In order to avoid this issue, a new mode matching method is used in [193] by electrostatically detuning the resonant frequency of the secondary mode of vibration until the amplitude of the secondary motion is maximized. Like the method in [3,22], a bias voltage is increased until nominal spring softening is increased to allow the sense frequency to be equal to the driving frequency. A difference is that the maximum is performed by using a perturbation-based extremum-seeking controller. The automatic mode matching method has an obvious advantage of real time tuning.

#### 3.3.3. Temperature Control and Compensation

**Figure 39.**
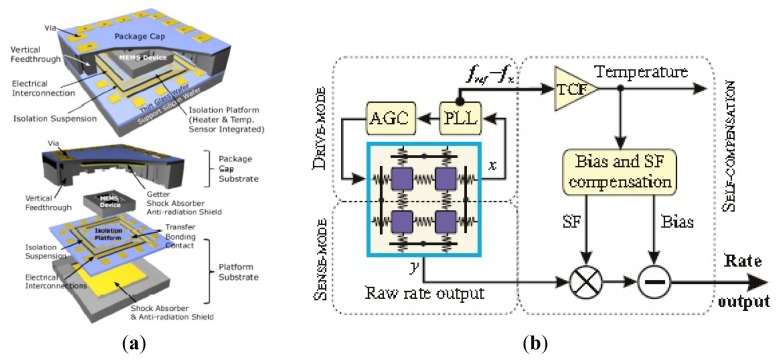
(**a**) University of Michigan vacuum packaging technology. (**b**) UC Irvine temperature compensation.

The working environment has a vital influence on the performance of a micromachined gyroscope, especially the temperature dependency of the bias and scale factor. The temperature drift error of a micromachined gyroscope may come from its mechanical structure or its control circuitry. The tests on temperature-dependent characteristics in [194] have proved that the temperature affects the gyroscope in two ways, through changing the resonant frequency and the resonator Q-factor. The analysis in [195] also illustrated that the thermal expansion caused by temperature can change gyroscope performance through changing structure dimensions. Thus, the temperature effect must be decreased to achieve a high precision gyroscope. In order to increase the robustness of the sensors against temperature fluctuations, structure and package optimization which is insensitive to the temperature is an effective method [196–198]. A vacuum packaging technology for environment-resistant MEMS devices as shown in Figure 39a was presented by a University of Michigan group [199]. The package structure consists of a platform substrate which provides thermal and mechanical isolation using suspensions, a supported MEMS device and flip-chip attached on the platform, and a package cap incorporating vertical signal feedthroughs and providing final vacuum encapsulation. A heater and a temperature sensor are located on the isolation platform to keep the device at a constant temperature higher than the maximum environmental temperature. The experimental drive frequency stays within 0.96 ppm/°C from −30 °C to 70 °C when the oven-control set-temperature is fixed at 80 °C. This package is capable of maintaining a long-term stable vacuum package, and provides robust vertical feedthroughs. The power consumption is less than 33 mW for oven-control at 80 °C.

On the other hand, temperature compensation by control circuit (on- or off-chip) has been another effective way. The temperature control provides the constant temperature environment to the gyroscope while the temperature compensation can measure the temperature effect and then provide possibility by electronic circuits externally or by software correction through onboard processors [200].

The bias drift compensation method is when the performance indexes changes with temperature are tested and then their relationship is modeled by different algorithms such as the linear function, least-squares method, neural network, Stepwise Regression Method, *etc.* In [201,202], a back propagation (BP) neural network is used to predict the temperature drift of a MEMS gyroscope and compensate it. This algorithm has the advantages of nonlinear fitting and mode identification capability, regardless of the mathematical model of the sensors and various nonlinear factors. In [203], linear fitting of segmenting linear functions is applied to eliminate the nonlinear temperature drift. This fitting method is only used in that specific situation. A stepwise linear regression method is used by the National University of Defense Technology in [204] for the output drift compensation of gyroscopes based on natural frequency. The gyroscope output is compensated through the natural frequency of the resonator instead of the temperature itself. Researchers from UC Irvine presented a long-term bias drift compensation algorithm for high quality factor (Q-factor) MEMS rate gyroscopes using real-time temperature self-sensing in 2013 [205], as seen in Figure 39b. The gyroscope drive mode is controlled by a PLL and an AGC loop and the open-loop sense mode is susceptible to temperature variations. To compensate the scale-factor and bias drifts influenced by temperature, the relationship between the silicon resonator frequency and temperature should be measured first. The test results show that the resonant frequency changes linearly with the temperature. Thus, the temperature is obtained through the Temperature Coefficient of Frequency (TFC) when the frequency changes. Once the instantaneous temperature value is obtained, the scale-factor and bias drifts are both compensated in real-time. This frequency-based measurement of temperature has the advantage of better stability over the amplitude-based measurements.

**Figure 40.**
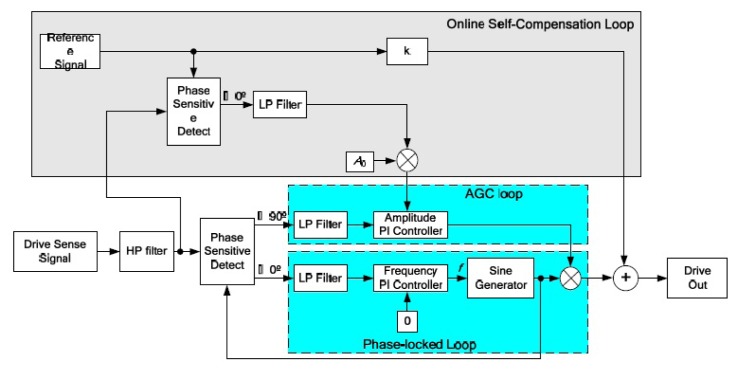
Scale factor compensation.

Figure 40 shows an online self-compensation system for micromachined gyroscopes to eliminate the scale factor drift due to the temperature influence [206]. As analyzed in the paper, when a gyroscope is working in primary close loop and secondary force rebalance loop, the main scale factor errors come from the influence of detection circuit error effected on the driving amplitude and driving nature frequency. A reference signal is added into the normal drive signal for observing the drift of Young's modulus and the detection circuit error. The amplitude of reference signal is detected by the phase sensitive detector using a reference signal and drive sense signal. The desired value of the AGC loop is adjusted according to the signal amplitude. Before compensation, the scale factor drift is −3.5% to 5.2% over the temperature range of −45 °C to +80 °C, while the scale factor drift decreases to −0.009% to 0.15% after compensation.

#### 3.3.4. Quadrature Compensation

As we know, the quadrature error is one of the most important error sources which influence the precision of gyroscopes. The mechanical quadrature signal may be so serious that it could be larger than the Coriolis signal, as analyzed in [207], that the amplitude of the Coriolis signal is so small in practical cases. Moreover, the required phase accuracy in the demodulation can also be strictly required. The quadrature error is mainly caused by the process imperfections such as mechanical imbalances and misalignments. It is theoretically analyzed through the equations of motion of a resonant gyroscope in [208]. It shows that there are two types of errors caused by the nondiagonal stiffness and damping coefficient matrices. The nondiagonal stiffness matrix causes the quadrature error, and the nondiagonal damping leads to the errors in phase. The quadrature signal is proportional to the primary mode displacement, while the Coriolis signal is proportional to the drive mode velocity. Thus, the phase difference between the Coriolis signal and the quadrature signal is 90 degrees theoretically. As a result, the quadrature error can be rejected by using a phase-sensitive demodulation. However, even very small phase errors will result in an unbearable offset because the amplitude of the quadrature error can be much larger than the Coriolis signal, therefore, the quadrature compensation is needed to improve the gyroscope precision.

**Figure 41.**
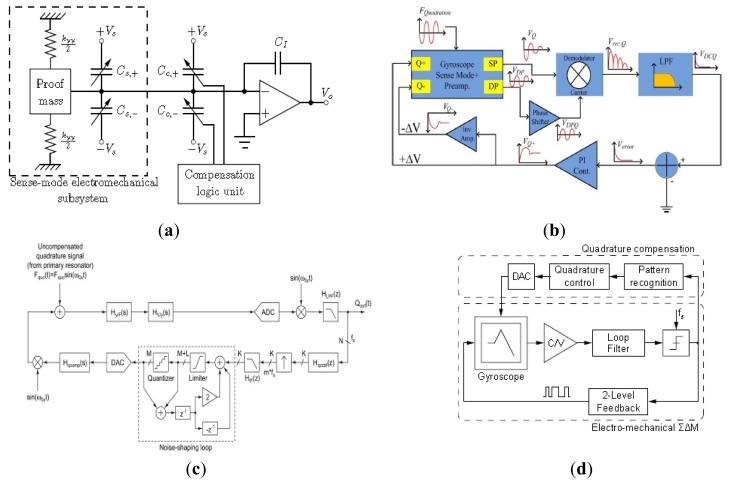
(**a**) Open loop charge-based compensation of the quadrature error. (**b**) Block diagram of the quadrature control electronics. (**c**) A feedback loop with ΣΔ DAC for quadrature signal compensation. (**d**) Electro-mechanical ΣΔ modulator with the proposed quadrature compensation.

There are several methods to handle the mechanical quadrature signal. First, they can be removed or reduced to a desired magnitude at the source of the sensor element using laser trimming or electrostatic “trimming” [209], but this is a time-consuming and expensive approach. Second, the quadrature error can be suppressed by careful design such as using special levers to improve the sensitivity of the suspension flexures [2]. These pure mechanical process techniques are appropriate because it solves the problem at the source and takes less die area than other servomechanism solutions. Further, a small quadrature signal requires the use of more precision phase detection for demodulation. Despite the mechanical method, the remaining quadrature signal still exists and needs to be cancelled by using circuit compensation. One approach is to use the electronic cancellation of the quadrature error by injecting an electronic signal with the same amplitude but opposite phase to the input port of the quadrature amplitude demodulator. Antonello *et al.* proposed an effective and simple method to generate the electronic signal in [210], as shown in Figure 41a. Note that only one pair is shown in the figure for simplification reasons. The capacitance arraies C_c_+ and C_c_− are controlled by compensation logic unit which is implemented in switched-capacitor technology to reach a finite amount of calibration levels. The output V_0_ is connected to the input stage of the quadrature error demodulator. Because of its inherent open loop structure, the proposed compensation technology is unable to provide robustness against variations of the quadrature error during the normal operation of the sensor. Another better approach that can eliminate the overall quadrature error by applying a DC voltage on the properly arranged electrodes in the sensor element.

Figure 41b shows the block diagram of the quadrature control electronics designed by Tatar *et al.* [211]. The quadrature error is eliminated by applying differential DC potentials on the mechanical electrodes of the sensor. Firstly, the quadrature signal is identified by a synchronous demodulator, then the output signal is rectified and passed through a low-pass filter (LPF) to get the amplitude information of the quadrature signal. Finally, the quadrature amplitude is compared to null, and the error output is fed to a PI controller to generate the DC potential ΔV which is differentially applied on the quadrature electrodes. This method greatly simplifies the controller design for the system.

Another two digital compensation methods for quadrature error are shown in Figure 41c and 41d, designed by Helsinki University of Technology [9] and University of Freiburg [212] respectively. In Figure 41c, the quadrature force is induced by the primary resonator movement and further excites the secondary resonator with the transfer function H_y/F_(s). The resulting signal passes through the displacement-to-voltage conversion H_V/y_(s), then it is converted to the digital domain through ADC. The quadrature error is demodulated into the amplitude voltage and the controller H_qcctrl_(s) is achieved in the FPGA. The analog DC voltage output through DAC is applied on the sensor to generate an electrostatic force by modulated at the operating frequency ω_0x_ in a proper phase. Q_out_(t) is the output quadrature signal. In this design, the resolution must be sufficient to keep the output signal stable. In order to increase the DAC resolution, the DAC is designed with ΣΔ Modulator that is preceded with an interpolator, interpolation filter H_IF_(z), and a noise-shaping loop (NL).

In Figure 41d, the gyroscope signal is first fed to a capacitive-to-voltage converter (C/V), the loop filter and ΣΔ modulator so that it can be converted into the digital domain. Then, the pattern recognition block analyses the output bit stream of the ΣΔ modulator in order to detect overload conditions. If an overload signal is detected and extracted, the output value of the quadrature control block would be readjusted. Finally, the digital quadrature control value is then transferred to the analog domain via the DAC and applied on the quadrature compensation electrodes of the sensor.

#### 3.3.5. Other Circuitry Technologies

##### Gyroscope arrays

**Figure 42.**
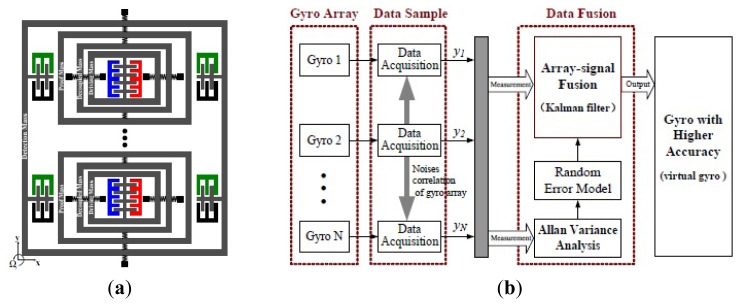
Gyroscope array. (**a**) Schematic of gyroscope array. (**b**) Digital process of gyroscope array.

In recent years, redundant MEMS gyroscopes have been integrated with GPS to improve navigation performance. Compared with a single poor gyroscope, multiple integrated gyroscopes can improve accuracy by means of appropriate signal processing. An array of MEMS gyroscopes proposed by Harbin Engineering University is shown in Figure 42a [213]. The gyroscope array combines two 4-DoF gyroscopes. The drive parts are set inside the whole gyroscope architecture, and the sense parts are set around the drive mode. This design makes it possible to combine several gyroscope units into a gyroscope array through sense modes of all the units. In order to improve the accuracy, rate signal modeling and the optimal filter must be designed carefully. The structure and principle of the gyroscope array is shown in Figure 42b. Several identical MEMS gyroscopes form a sensor array and the multiple data are collected by the data acquisition system. Then, they are used to quantify the random errors through noise modeling by the Allan variance. A Kalman filter (KF) based on first-order Markov process is designed to obtain optimal rate estimates in [214]. The experimental results indicate that an array with six gyroscopes which have an ARW noise of $6.2{}^{\circ}/\sqrt{\text{Hz}}$ and a bias drift of 54.14°/h could be combined into a rate signal with an ARW noise of $1.8{}^{\circ}/\sqrt{\text{Hz}}$ and a bias drift of 16.3°/h.

##### Self-calibration

Normally, the scale factor of a gyroscope will drift over time. To redefine the scale factor during use is troublesome. Self-calibration of a gyroscope is a valuable feature that has the advantages of simplifying operation, eliminating expenses and time consumption caused by mechanical calibration of the device using rate tables. Researchers from Tsinghua University have developed a new calibration procedure to determine the scale factor of a gyroscope without a turntable [172]. The force of gravity is used to deflect the movable masses in the gyroscope, which results in a corresponding angular rate input. The method only provides a constant angular rate which causes a deviation compared with the linear fitting through multi-dots. Georgia Tech scientists introduced another approach to self-calibration of Coriolis-based vibratory gyroscopes [215]. The effect of the Coriolis force on the device is mimicked by the application of a rotating excitation to the device drive and sense modes. Equivalently amplitude-modulated excitations are applied in the drive mode and sense mode. Then a small phase shift which is proportional to the input rate will be induced in the modulating envelope of the gyroscope response. The small phase shift replaced by DC voltage can be detected by circuitry technology. Compared with the method in [214], the equivalent input angular rate can be set by customer. However, this method must be applied to the mode-matched gyroscope.

##### Special technologies

Other technologies are widely used to improve the performance of a gyroscope, such as anti-shock, bias drift compensation, anti-acoustically noise, *etc.* Among the MVGs, multi-DoF or stopper is a very efficiently way to improve the robustness to the shock. A TFG with two level elastic stoppers designed by Zhou *et al.* can improve the shock resistance of ten thousands of g [29]. In order to find the shock impact, Li *et al.* from Aalto University presented an interesting experiment applying various shock loads on a three-axis MEMS gyroscope. They found that the major failure modes caused by shock are package and functional failures. The package failure is primarily because of the package deformation during the shock impacts. The functional failure is the result of stiction, particulate-induced blocking, internal collisions and fractures in the comb arm-finger structures [216]. Temperature will cause a nonlinear thermal bias drift. Hong proposed a nonlinear fuzzy compensation scheme to overcome the temperature-induced bias drift of the resonant rate sensor [217]. The nonlinearity of bias drift is represented by a Takagi–Sugeno fuzzy model over the entire range of the operating temperature. Then, the fuzzy model is directly used for compensation of nonlinear bias drift by subtracting the estimated output from the raw data. MEMS gyroscopes are also susceptible to acoustic noise when acoustic energy frequency components are close to the resonant frequency of the gyroscope [218]. The interesting investigation shows that high-power high-frequency content acoustic noise environments can deleteriously severely affect the performance of MEMS gyroscopes. Above all, the typical analog/digital circuits, special circuits and special technologies are discussed in detail. Their circuits and characteristics can be summarized and shown in Table 12.

## 4. Discussion and Future Prospects

As MEMS technology improves, MEMS gyroscopes will see wide application in the fields of national defense, industry, and other consumer products. The market and military both require the MEMS gyroscopes to be higher performance, lower cost, with higher integration, multi-functionality, smaller volume and stronger environmental tolerance. Among the MVGs, TFGs and VRGs have been reported remarkable performance with bias drifts of about 0.1°/h. However, their performance is limited currently because of the vibration structure. Moreover, the MVGs have not enough robustness to the poor environment, shock and other interferences. Despite of that, MVGs are still the most appropriate products in the commercial field because of their lower cost corresponding to precision. In some application fields, we focus more on the cost performance and perfect function other than its precision since the ultrahigh precision is sometimes not required. Therefore, how to lower the cost, integrate multi-functionality and improve the environment ability of MVGs are important topics for all researchers. Multi-DoF gyroscopes seem to have better robustness to harsh environments while there is still some time to solve the contradiction between bandwidth and sensitivity. Researchers will provide new technology innovation to solve this problem such as frequency modulation gyroscopes. Multi-axis gyroscopes are another development direction in MVGs. Commercial functions require the gyroscopes to be combined with accelerometers or compasses. Thus, the volume could be too large to be integrated on a chip. To solve the problem above, multi-axis gyroscopes have inherent advantages for integration with accelerometers for inertial measurement unit (IMU) applications.

PVGs are some of the simplest gyroscopes with robustness, wide measuring range and higher resistance to outer shocks and shaking without supporting mechanical structures. With fully symmetrical structures multi-axis detection is easy to achieve. Apart from the advantages above compared with MVGs, they have lower cost because they can work in atmospheric environments and have no special vacuum packaging requirements. These prominent advantages attract lots of researchers to focus on PVGs. However, the modes of a PVG are complicated so that the deformation is hard to detect. Performance of a pure PVG is relatively poor when used by itself in practical applications to date, but if we design a gyroscope with a special structure so that there are several simple modes for detection, the problem will be effectively solved. Another direction is to combine the PVGs with other gyroscopes such as piezoelectrically actuated MVGs, micro fluid gyroscopes, *etc.* As another solid gyroscope type, SAW gyroscopes have similar advantages as PVGs. Moreover, SAW gyroscopes are compatible with the standard IC process so that the cost will be lower. However, the performance of the gyroscopes is still rarely reported because of the intricate gyroscope principle, structure and metal dots. Using the wireless sense mode, there exists a problem that how to eliminate inherent insertion losses and ensure RF technology. The BAW gyroscope is also a solid gyroscope with the same advantages of robustness, wide measuring range and higher resistance to outer shocks and shakes. In addition, the resonance frequencies of BAW gyroscopes are extremely high from several to dozens of MHz, so the noise floor largely decreases, since it relies on the increase in the mass, excitation of the driving amplitude and resonant frequency. For a low frequency gyroscope such as MVGs, PVGs *etc.*, in order to decrease the noise floor, they increase either the mass or driving amplitude so that it is difficult to achieve relatively low power and small size. High resonant frequency leads to the challenge of signal processing at the same time.

Suspended gyroscopes including MESGs and MSGs also have no supporting mechanical structures so they are insensitive to fabrication errors, stresses and temperature variations resulting in higher performance. Moreover, a suspended gyroscope can easily measure multi-angular rates and acceleration so it is effective to reduce the size and cost of integrated IMUs. However, the performance depends on the rotation speed which is constrained by the suspended rotor material strength. For MESGs, they need a complicated feedback control circuit or a dynamically tuned circuit to control the rotor. Moreover, ultrahigh vacuum packaging is essential to get a high rotation speed while the heat caused by the eddy is hard to diffuse. How to increase the rotation speed and decrease the heat is a current task. The special technologies such as temperature control and compensation seem to be an attractive method. A diamagnetic suspended rotor can be suspended stably by itself so it does not have the problems of MESGs mentioned above. A MSG has the advantages of simple structure, no energy input and no feedback control circuit so that it can attain a high rotation speed. On the contrary, a MSG needs a permanent magnet or a coil to suspend the rotor that the volume is too large to be integrated on a chip.

Micro fiber optic gyroscopes and micro atom gyroscopes are the most promising gyroscopes with high precision, long life time and robustness to the environment. Compared with other gyroscopes, MOEMS gyroscopes can reach higher sensitivity without any moving parts and vacuum packaging. Through the efforts of more than 20 years of work, MOEMS gyroscopes have achieved a great deal of progress. To date, the precision of MOEMS gyroscopes have achieved several degrees per hour and there is a lot of room for further improvement. There are mainly four factors limiting the gyroscope performance, including the Rayleigh backscattering, Kerr, Faraday and thermal effects. New structures, materials, and ways of signal processing are under study to develop low loss waveguides, narrow band light or noise suppression. The atom gyroscope is one of the most potential high precision gyroscopes whose sensitivity can reach an order of magnitude of 10^−10^ in large scale. For NMRGs, the tasks are how to maintain the magneto-optical trap and how to export atoms arbitrarily along constant orientation. For atom interference gyroscopes, they have a potential sensitivity 10^10^ greater than optical interference gyroscopes. However, the disorder motion of an atom causes a significant degradation of performance. These problems must be solved urgently to realize the potential performance.

Although the fiber optic or atom angular velocity sensors provide extremely precise information, the price is too high to be widely used in the consumer field. MVGs are the main products on the market because of their low cost and long lifetimes. Micro fluid gyroscopes also have potential applications in the consumer field with their advantages of low cost, and enough robustness against external impacts. However, to date, the desired precision has been a hard to reach requirement for MVGs because the accuracy of the temperature sensed through thermistors is not high. Moreover, the fluid or gas flows are sometimes irregular, especially when a shock is applied on the gyroscope. The micro fluid gyroscope is only suitable for some fields which do not require high precision. The emergence of atom gyroscopes, nano-gyroscopes and molecular gyroscopes play an important role in gyroscope theory innovation. These gyroscopes can be smaller with higher integration compared with other gyroscopes. Although there is no detailed information about the performance of these gyroscopes, new kinds of gyroscopes are worthy of investigation in the future. Some typical kinds of micromachined gyroscope performances, characteristics and applications are listed in Table 13.

As another important part of MEMS gyroscope systems, the circuitry can also greatly influence the performance. For pure analog circuits, while they bring additional noise, temperature drift and poor stability, they are easy to achieve. Moreover, it is not realistic to achieve complex control algorithms. On the contrary, digital circuits have the advantages of low power consumption, high integration, high stability, easy debugging, self-processing, *etc.* In order to adjust to the poor environment for some special situations, temperature control and compensation is useful, especially chip-level temperature compensation with small volume. For a high performance gyroscope, low noise and high integration are the primary choices. As a result, ASIC technologies on a chip with MEMS gyroscopes, also evolved into the mixed analog and digital system on chip (SOC), would be a main trend for high precision, small volume, low power consumption and low cost. To decrease the influence of analog circuits with high noise, large temperature drift and inflexibility, sigma delta interface circuits provide a perfect method of achieving hybrid digitalized circuit systems which are easy to adapt for subsequent self-testing, self calibration, or other intelligent functions. These advantages make digitalization technology prevail. On the other hand, the innovation of new kinds of control circuitry and novel control algorithms is as important as the gyroscope structure innovation.

**Figure 43.**
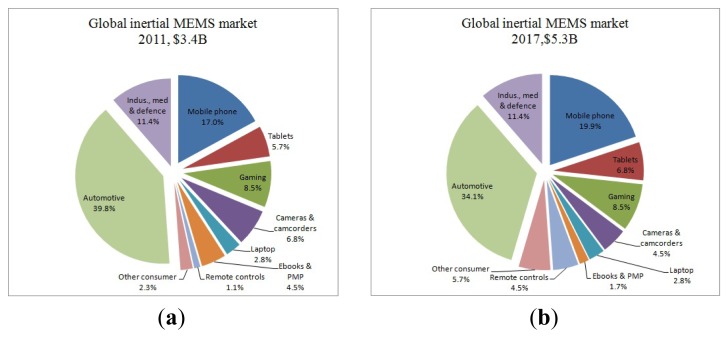

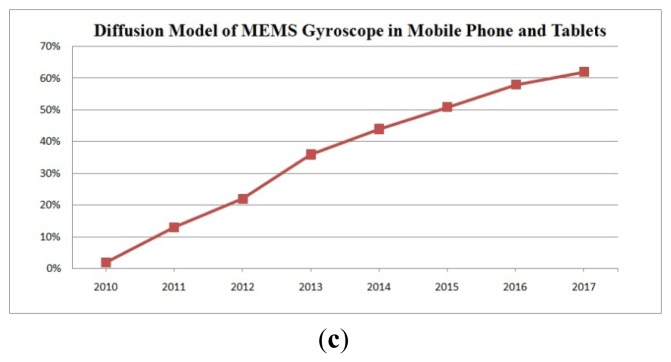
Inertial MEMS products situation. (**a**) Global inertial MEMS market in 2011. (**b**) Global inertial MEMS market in 2017. (**c**) Diffusion of MEMS gyroscopes in mobile phone and tablets.

MEMS products including gyroscopes, accelerometers *etc.* are widely used in the national defense, industries, mobile phones, automotive and other fields. An interesting report presented by Yole Development company shows that the MEMS sensors will maintain strong growth in the future [219]. The market situation proved by Yole Development in 2012 is shown in Figure 43a. The total revenue of the inertial MEMS including gyroscope, accelerometers and magnetometer in 2011 had broken through $3.4 billion and the record will break through $5.3 billion in 2017, prospectively, as seen in Figure 43b. The largest application field of the MEMS inertia sensors is automotive and it is continuing to grow in the future. MEMS Gyroscopes in consumer field such as mobile phone and tablets will account for more and more proportion. The diffusion model of MEMS gyroscope in mobile phone and tablets is shown in Figure 43c. In 2010, MEMS gyroscopes were used by only 2% of mobile phone and tablets, while the proportion will increase rapidly to about 60% in 2017, prospectively.

There are lots of manufacturers focusing on MEMS gyroscopes, such as STMicroelectronics, InvenSense, ADI, Silicon Sensing, BEI Technologies, Sensors, *etc.* ST is one of the largest MEMS gyroscope suppliers [220]. Their MEMS gyroscope revenue reached about $260 million in 2011 and it is still growing. Their products include analog inertial devices such as the LPR403AL, LPY403AL, LY3100ALH *etc.*, and digital inertial devices such as the A3G4250D, L3G4200D *etc.* Especially, the gyroscopes offer superior stability over time and temperature, with a resolution lower than $0.03{}^{\circ}/\text{s}/\sqrt{\text{Hz}}$ for zero-rate level which guarantees the level of accuracy required by the most advanced motion-based applications. Their 3-axis gyroscopes utilize a single sensing structure for motion measurement along all three orthogonal axes, which is the simplest solution on the market. Besides, ST's angular rate sensors have already been used in mobile phones, tablets, 3D pointers, game consoles, digital cameras and many other devices. InvenSense is one of the largest motion-tracking device suppliers [221]. InvenSense products cover nine-axis motion-tracking devices (three gyroscopes, three accelerometers and three compasses) including the MPU-9250, MPU-9150 *etc*, six-axis MEMS motion processors (3-axis gyroscope and 3-axis accelerometer) including the MPU-61N1, MPU-6000 *etc.* and MEMS gyroscopes including the triple-axis ITG-31N1 gyroscope, the dual-axis IXZ-2020 gyroscope, and single-axis ISZ-500 gyroscope, *etc.* In 2011, InvenSense's revenue was about $80 million, in which about 10% was for motion-tracking devices and MEMS motion processors and the proportion is growing. InvenSense's motion-tracking devices such as the MPU-9250, MPU-9150 *etc.* are rapidly becoming a key function in many consumer electronic devices including smart phones, tablets, gaming consoles, and smart TVs. ADI Company is one of the oldest MEMS gyroscope suppliers [222]. ADI's MEMS gyroscopes first offer a range of MEMS sensor and signal conditioning integration on chips for consumer field. ADI's gyroscope products have the advantages of high immunity to shock, vibrations and linear acceleration. The ADXRS series gyroscopes such as the ADXRS649, ADXRS800 *etc.* have 2000 *g* power shock survivability. ADI also supplies many kinds of MEMS inertial measurement units, such as the 4-DoF inertial sensor ADIS16300 (a yaw rate gyroscope and tri-axis accelerometer), 6-DoF inertial sensor ADIS16485 (a tri-axis gyroscope and a tri-axis accelerometer), 10-DoF inertial sensor ADIS16488 (a tri-axis gyroscope, a tri-axis accelerometer, tri-axis magnetometer and pressure sensor), *etc.* They are available for various applications. Silicon Sensing has produced a wide range of single-axis silicon MEMS gyroscope sensors [223]. Their different performance gyroscopes suit most applications where customers value product functionality, performance and integrity, and expert technical support. Silicon Sensing provides fiber optic gyroscope levels of performance in high shock and vibration environments. Their MEMS gyroscope products include the CRG20, CRH01, SIRRS01, and the CRS series in which CRS39 shows a ultrahigh precision with a bias drift of 0.2°/h and 0.14°/s bias drift over temperature.

## 5. Conclusions

In this review, various typical micro gyroscope structures with different materials and fabrication as well as circuitry technologies are discussed in detail. As illustrated above, the new structures, materials and fabrication technologies are being constantly updated to improve the performance, including the precision and environment capability. In theory, the ideal micro gyroscopes should satisfy the basic standards of low cost and miniaturization. In order to meet different application requirements, there are two directions for further development from our point of view. One trend is to pursue even higher precision. Since it will not be easy to achieve breakthroughs in the precision area with traditional micro gyroscopes in a short time, both new structures with special materials and fabrication technology are necessary. Novel gyroscopes with creative principles, such as the micro atom gyroscope, nano-gyroscope, micro fluid gyroscope and other hybrid gyroscopes *etc.*, should be explored in depth. The other trend is to realize the multi-axis or multi-DoF functional gyroscopes in highly integrated MIMU with certain environmental adaptability despite of the precision in the future, considering the consumer electronic market is huge. Surely, the matched circuit technologies or measurement methods should be synchronously improved with the gyroscopes.

## Figures and Tables

**Table 1.** The improvement of the Georgia Tech TFG.

**Parameter**	**2004**	**2006**	**2008**
Substrate	40 μm SOI	40 μm SOI	60 μm SOI
Quality factor	2000	40,000	36,000
Max. Scale Factor	1.25 mV/°/s	24.2 mV/°/s	88 mV/°/s
Bias Drift	>5°/h	1°/h	0.1°/h
Bandwidth	12 Hz	1 Hz	1 Hz–10 Hz
Brownian Noise	$20{}^{\circ}/\text{h}/\sqrt{\text{Hz}}$	$0.5{}^{\circ}/\text{h}/\sqrt{\text{Hz}}$	$0.18{}^{\circ}/\text{h}/\sqrt{\text{Hz}}$

**Table 2.** The summary of TFGs.

**Institute**	**Time**	**Fabrication**	**Drive/Sense**	**Detected Axis**	**References**
SIMIT and SJTU	2005,2006	Bulk micromachining	Electromagnetical/ capacitance	Z	[16,17]
SIMIT and TJU	2010, 2013	Bulk micromachining	Electrostatic/ capacitance	Z	[18,29]
PKU	2010	SOG	Electrostatic/ capacitance	Lateral	[19,20]
Georgia Tech	2004–2009	SOI	Electrostatic/ capacitance	Z	[21–25]
CEA-LETI	2013	SOI	Electrostatic/ capacitance	X/Y/Z	[26]
HSG-IMIT	2007	SOI	Electrostatic/ capacitance	X/Y/Z	[27]
NUDT	2012	Quartz anisotropic wet etching	Electrostatic/ shear stress	Z	[28]

**Table 3.** The summary of decoupled gyroscopes.

**Institute**	**Time**	**Decoupled Style**	**Fabrication**	**Drive/Sense**	**Detected Axis**	**Reference**
PKU	2009	Double decoupling	SOG	Electrostatic/ capacitance	Lateral	[30,31]
HSG-IMIT	2003	Double decoupling	Bulk micromachining	Electrostatic/ capacitance	Z	[32]
UC Irvine	2005	Double decoupling	Bulk micromachining	Electrostatic/ capacitance	Z	[33]
Middle East Tech	2002–2008	Double decoupling	Surface/bulk micromachining	Electrostatic/ capacitance	Z	[34–39]
Korean universities	2003,2008	Single decoupling	Surface micromachining	Electrostatic/ capacitance	Lateral	[40,41]
NTU	2012	Double decoupling	MetalMUMP	Electrostatic/ capacitance	Z	[42]
Saarland University	2012	Double decoupling	Surface micromachining	Electrostatic/ capacitance	Z	[43]

**Table 4.** The summary of decoupled gyroscopes.

**Institute**	**Time**	**Fabrication**	**Gimbal** **Amount**	**Drive/Sense**	**Detection** **Axis**	**Reference**
Draper	1991	Bulk micromachining	Double	Electrostatic/ capacitance	Z	[44]
HSG-IMIT	1999	Bosch Foundry process	Double	Electrostatic/ capacitance	X	[46]
SIMIT	2002	Bulk micromachining	Single	Electrostatic/ capacitance	Z	[47]
University of Hyogo	2003	SOI	Double	Electromagnetic/ potential	Z	[48]

**Table 5.** The summary of VRGs.

**Institute**	**Time**	**Fabrication**	**Materials**	**Drive/Sense**	**Detected Axis**	**References**
UMich	1998	Bulk/surface micromachining	Ploysilicon	Electrostatic/ capacitance	Z	[49]
CAS	2010, 2012	Bulk micromachining	Single crystal silicon	Electromagnetic/ inductive	Z	[52,53]
	2010	HARPSS	Single crystal silicon	Electrostatic/ capacitance	Z	[54]
Georgia Tech	2005	HARPSS	Polysilicon	Electrostatic/ capacitance	Z	[55]
NCL	2005, 2011	Bulk micromachining	Polysilicon	Electrostatic/ capacitance	Z	[56,57]

**Table 6.** The summary of multi-DoF gyroscopes.

**Institute**	**Time**	**Fabrication**	**DoF**	**Detected Axis**	**References**
UC Irvine	2006–2011	Bulk micromachining	2-DoF sense, 1-DoF drive	Z	[59–62]
	2011	SOI	1-DoF sense, 2-DoF drive	Z	[63]
	2007	SOI	4-DoF sense, 2-DoF drive	Z	[65]
CMU	2009	SOG	2-DoF sense, 1-DoF drive	Z	[64]
HEU	2012	Bulk micromachining	2-DoF sense, 2-DoF drive	Z	[66]
Pakistan	2011	MetalMUMP	1-DoF sense, 2-DoF drive	Z	[67]

**Table 7.** The summary of multi-axis MVGs.

**Institute**	**Time**	**Fabrication**	**Axis**	**Materials**	**Detected Axis**	**References**
Berkeley	1997	Surface micromachining	2	Polysilicon	X/Y	[68]
Georgia Tech	2011	HARPSS	2	Polysilicon	X/Y	[69]
NTHU	2006	Surface micromachining	2	Polysilicon	X/Y	[70]
NTU	2009	CMOS-MEMS	2	Polysilicon	X/Y	[3]
NCKU	2012	Bulk micromachining	2	Silicon, glass	X/Z	[71]
	2008–2010	Bulk micromachining	3	Silicon, glass	X/Y/Z	[72–74]

**Table 8.** The summary of SAW gyroscopes.

**Institute**	**Time**	**Fabrication**	**Frequency(MHz)**	**Substrate**	**Materials**	**References**
Ajou University	2009–2012	Lift-off	80	128° YX LiNbO_3_	Al,Au	[87–89]
CAS	2010	Evaporation and wet etching	80	128° YX LiNbO_3_	Al	[90]
NUDT	2009	Evaporation and wet etching	95.9	128° YX LiNbO_3_	Al	[92]

**Table 9.** The summary of MESG gyroscopes.

**Institute**	**Time**	**Fabrication**	**Materials**	**Detected Axis**	**References**
Tohoku	2003	DRIE	Silicon, glass	X/Y/3-axis acceleration	[100]
Southampton	2001–2006	Bulk micromachining	Silicon, glass	X/Y	[101–103]
SJTU	2009–2010	UV-LIGA	Nickel, glass	X/Y/3-axis acceleration	[104–107]
THU	2012	Bulk micromachining	Silicon, glass	X/Y	[108]

**Table 10.** The summary of MSG gyroscopes.

**Institute**	**Time**	**Fabrication**	**Materials**	**Rotation Speed (rpm)**	**References**
NTU	2000	Bulk micromachining	Si, Al	1000	[110]
SJTU	2006	Bulk micromachining	Al_2_O_3_, Al, Cu	3035	[111]
	2008	Surface micromachining	Silicon, Al_2_O_3_	Over10 in the air	[112]
	2010	Bulk micromachining	Glass, Cu, Ni,	Changeable	[113]
UEST	2009	Bulk micromachining	/	12,000	[114]
NCKU	2009	Surface micromachining	Cr, Au, Cu	Sin, 6250 Hz	[115]

**Table 11.** The summary of micro fluid gyroscopes.

**Institute**	**Time**	**Size**	**Materials**	**Drive/Sense**	**Detected Axis**	**References**
Keio University	2008	40 × 60 × t7 mm^3^	Liquid	Jet/thermistor	Z	[134,135]
TSCL, RU	2004	400 × 4 × 2 um^3^	Liquid	Piezoelectric/ thermistor	X/Y	[136]
RU	2013	Φ16× 1 mm^3^	Liquid	Piezoelectric/ thermistor	X/Y	[137]
HUST	2006	8 × 0.4 × 0.25 mm^3^	Liquid	Thermal/ thermistor	X/Y	[138]
NUST, SFU	2013	2020 × 990 × 315 um^3^	Gas	Thermal/ thermistor	Z	[139,140]
NPU	2013	Φ20 × 7 mm^3^	Gas	Jet/thermistor	X/Y/Z	[141]

**Table 12.** Summary of circuits used in micromachined gyroscopes.

**Categories**	**Circuits**		**Characteristics**
Typical analog circuits	Drive circuits	AC sine wave	Implemented Easily and simply, need multi-devices, may have large noise, drift, and clumsy

		AC square wave	

		DC sine wave	

		DC square wave	

	Sense circuits	Open-loop	Easy realization, reducing additional noise and high efficiency, poor stability, limited bandwidth

		Closed-loop	Complex control circuits, better stability, can achieve mode matching, compensation *etc*

Typical digital circuits	Drive circuits		Implemented on the same chip based on DSP or FPGA, low power consumption, high integration, high stability, easy debugging, self-processing

	Sense circuits		

Special circuits	Sigma delta		Implemented by closed-loop control in the digital domain, good dynamic range and stability, high precision and integration

	Mode matching		Ultrahigh sensitivity, high precision, poor bandwidth

	Temperature control and compensation		Decrease the temperature drift largely, high precision, good robustness, extra circuits

	Quadrature compensation		High precision, complex control system, high cost

Special technologies	Gyroscope array		Low cost, high efficiency, complex signal process, large volume

	Self-calibration		Simplify operation, reduce expense and time consumption, applied to some special gyroscopes

**Table 13.** Comparison of some typical kinds of micromachined gyroscopes.

**Categories**	**Performance** **(at Present)**	**Characteristics** **(in Theory)**	**Application**
MVG	Better level, bias drift has reached sub-degree per hour	Ease to control, high stability, have suspending springs and proof masses, poor robustness, mode mismatched, usually vacuum packaged	Mature at present, the most widely used in the micromachined gyroscope market

PVG	Lowest	Good robustness, wide measuring range and higher resistance to shock and shake, can work in atmospheric environment, poor stability, hard in signal extraction	Less used at present

SAW gyroscope	Lower	Good robustness, very wide measuring range, low power consumption, low sensitivity, compatible with standard IC process, complex measurement circuit	Less used at present, but have good application prospect because of low power consumption and wireless circuitry

BAW gyroscope	Good level, bias drift can reach tens of degree per hour	High resonant frequency and Q-factor, very low noise floor, low cost, sustaining electronics noise and high bandwidth requirement due to the high frequency	Less used at present, but have good application prospect

MESG	Middle level, bias drift can reach dozens of degree per hour	Long life without mechanical friction, insensitive to fabrication tolerances, mode coupling, high cost, complex circuitry	Have some application at present, and will have best application prospect

MSG	Low	Simple structure, no energy input and simple control circuit, very little mechanical friction, insensitive to fabrication tolerances, can work at atmosphere, mode coupling, poor stability	Technology is still not mature enough, less used at present

MFOG	Several degree per hour	High precision, long life and robustness, wide dynamic range, strict light source and signal process, high cost	Technology is still not mature enough, have improvement space

MAG	Still developed	Potential high sensitivity, ultra-small volume, ultra-low power consumption, hard detection, high requirement for measurement instrument	Technology is still not mature enough, have improvement space

MFG	Low	Long life, good robustness, ultra-low cost, low precision	Have improvement space in low precision fields

## Abbreviation Index

*MVG*: Micromachined vibrating gyroscope
*PVG*: Piezoelectric vibrating gyroscope
*SAW*: Surface acoustic wave
*BAW*: Bulk acoustic wave
*MESG*: Micromachined electrostatically suspended gyroscope
*MSG*: Magnetically suspended gyroscope
*LIGA*: Lithographie Galvanoformung Abformung
*CW*: Clockwise
*CCW*: Counterclockwise
*NMRG*: Nuclear magnetic resonance gyroscope
*TFG*: Tuning fork gyroscope
*M2-TFG*: Mode-matched tuning fork gyroscope
*SOI*: Silicon-on-insulator
*ARW*: Angle random walk
*SNR*: Signal-to-noise ratio
*ZRO*: Zero-rate-output
*MARS-RR*: Micromachined angular rate sensor with two rotary Oscillation modes
*DRIE*: Deep reactive ion etching
*VRG*: Vibrating ring gyroscope
*HARPSS*: High aspect-ratio combined poly and single-crystal silicon MEMS technology
*SCS*: Single-crystal silicon
*RSG*: Resonating star gyroscope
*DVA*: Dynamic vibration absorber
*MetalMUMP*: Metal-Multi User MEMS Process
*DTFG*: Dual-Axis TFG
*SOG*: Silicon-on-glass
*PMMG*: Piezoelectric micromachined modal gyroscope
*IDT*: Interdigital transducer
*TED*: Thermoelastic damping
*PM*: Permanent magnet
*FOG*: Fiber optic gyroscope
*IFOG*: Interferometer fiber optic gyroscope
*MOEMS*: Micro optical electromechanical system
*RLG*: Ring laser gyroscope
*MOEMS*: Micro optical electromechanical system
*MQW*: Multiple quantum-well
*SRL*: Semiconductor ring laser
*PBF*: Photonic-bandgap fiber
*NMRG*: Nuclear magnetic resonance gyroscope
*AIG*: Atom interferometry gyroscope
*ASG*: Atom spin gyroscope
*ECF*: Electro-conjugate fluid
*DFTB*: Density-functional-based tight-binding
*HRG*: Hemispherical Resonator Gyroscope
*MHG*: Microelectromechanical Hybrid Gyroscope
*CNT*: Carbon nanotubes
*FIB*: Focused Ion Beam
*AM*: Amplitude modulation
*FM*: Frequency modulation
*QMG*: Quadruple Mass Gyroscope
*DETF*: Double-ended tuning fork
*AGC*: Automatic gain control
*PLL*: Phase locked loop
*VGA*: Variable-gain amplifier
*PI*: Proportional-integral
*LPF*: Low-pass filter
*LIA*: Lock-in amplifier
*LMSD*: Least mean square demodulation
*LMS*: Filter-least mean square
*DDS*: Direct digital frequency synthesizer
*CORDIC*: Coordinate Rotate Digital Computer
*NCO*: Numerical control oscillator
*ADC*: Analog to digital converter
*NTF*: The noise transfer function
*DSP*: Digital signal processing
*CSA*: Charge-sensitive amplifier
*HV*: High-voltage
*DAC*: Digital-to-analog converter
*HRZ*: Half-return-zero
*RZ*: Return-zero
*NL*: Noise-shaping loop
*C*/*V*: Capacitive-to-voltage converter
*IMU*: Inertial measurement unit
